# Dual inhibition of EZH2 and G9A/GLP histone methyltransferases by HKMTI-1-005 promotes differentiation of acute myeloid leukemia cells

**DOI:** 10.3389/fcell.2023.1076458

**Published:** 2023-03-23

**Authors:** Y. Sbirkov, T. Schenk, C. Kwok, S. Stengel, R. Brown, G. Brown, L. Chesler, A. Zelent, M. J. Fuchter, K. Petrie

**Affiliations:** ^1^ Division of Clinical Studies, The Institute of Cancer Research, London, United Kingdom; ^2^ Department of Medical Biology, Medical University of Plovdiv, Plovdiv, Bulgaria; ^3^ Research Institute at Medical University of Plovdiv, Plovdiv, Bulgaria; ^4^ Department of Hematology and Medical Oncology, Clinic of Internal Medicine II, Jena University Hospital, Jena, Germany; ^5^ Institute of Molecular Cell Biology, CMB, Jena University Hospital, Jena, Germany; ^6^ Division of Gastroenterology, Hepatology and Infectious Diseases, Department of Internal Medicine IV, Jena University Hospital, Jena, Germany; ^7^ Department of Surgery and Cancer, Imperial College London, London, United Kingdom; ^8^ Institute of Clinical Sciences, School of Biomedical Sciences, College of Medical and Dental Sciences, University of Birmingham, Birmingham, United Kingdom; ^9^ Department of Molecular Biology, Institute of Genetics and Animal Biotechnology, Polish Academy of Science, Magdalenka, Poland; ^10^ Department of Chemistry, Molecular Sciences Research Hub, Imperial College London, White City Campus, London, United Kingdom; ^11^ School of Medicine, Faculty of Health Sciences and Wellbeing, University of Sunderland, Sunderland, United Kingdom

**Keywords:** EZH2/KMT6A, G9A/EHMT2, GLP/EHMT1, differentiation, acute myeloid leukemia (AML), all-*trans* retinoic acid (ATRA), RARα, HKMTI-1-005

## Abstract

All-*trans*-retinoic acid (ATRA)-based differentiation therapy of acute promyelocytic leukemia (APL) represents one of the most clinically effective examples of precision medicine and the first example of targeted oncoprotein degradation. The success of ATRA in APL, however, remains to be translated to non-APL acute myeloid leukemia (AML). We previously showed that aberrant histone modifications, including histone H3 lysine 4 (H3K4) and lysine 27 (H3K27) methylation, were associated with this lack of response and that epigenetic therapy with small molecule inhibitors of the H3K4 demethylase LSD1/KDM1A could reprogram AML cells to respond to ATRA. Serving as the enzymatic component of Polycomb Repressive Complex 2, EZH2/KMT6A methyltransferase plays a critical role in normal hematopoiesis by affecting the balance between self-renewal and differentiation. The canonical function of EZH2 is methylation of H3K27, although important non-canonical roles have recently been described. EZH2 mutation or deregulated expression has been conclusively demonstrated in the pathogenesis of AML and response to treatment, thus making it an attractive therapeutic target. In this study, we therefore investigated whether inhibition of EZH2 might also improve the response of non-APL AML cells to ATRA-based therapy. We focused on GSK-343, a pyridone-containing S-adenosyl-L-methionine cofactor-competitive EZH2 inhibitor that is representative of its class, and HKMTI-1-005, a substrate-competitive dual inhibitor targeting EZH2 and the closely related G9A/GLP H3K9 methyltransferases. We found that treatment with HKMTI-1-005 phenocopied *EZH2* knockdown and was more effective in inducing differentiation than GSK-343, despite the efficacy of GSK-343 in terms of abolishing H3K27 trimethylation. Furthermore, transcriptomic analysis revealed that in contrast to treatment with GSK-343, HKMTI-1-005 upregulated the expression of differentiation pathway genes with and without ATRA, while downregulating genes associated with a hematopoietic stem cell phenotype. These results pointed to a non-canonical role for EZH2, which was supported by the finding that EZH2 associates with the master regulator of myeloid differentiation, RARα, in an ATRA-dependent manner that was enhanced by HKMTI-1-005, possibly playing a role in co-regulator complex exchange during transcriptional activation. In summary, our results strongly suggest that addition of HKMTI-1-005 to ATRA is a new therapeutic approach against AML that warrants further investigation.

## 1 Introduction

In the 40 years since a role for altered epigenetics in cancer by way of aberrant global DNA hypomethylation was first demonstrated (Feinberg and Vogelstein, 1983), that cancer cells under epigenetic reprogramming has become an established principle and was added recently to the third iteration of the “Hallmarks of Cancer” (Hanahan, 2022). The reversible nature of epigenetic modifications makes them excellent therapeutic targets (Cheng et al., 2019), exemplified by our study using tranylcypromine (TCP), an inhibitor of the histone H3 lysine 4 (H3K4) demethylase LSD1 (KDM1A), to demonstrate that drug-induced epigenetic remodeling could reprogram acute myeloid leukemia (AML) cells to respond to all-*trans* retinoic acid (ATRA, tretinoin)-based therapy (Schenk et al., 2012). Following on from this study, clinical trials using TCP (Wass et al., 2021; Lübbert et al., 2022) and a number of other LSD1 inhibitors are underway (Fang et al., 2019). Acute myeloid leukemia (AML) is the most common acute hematological malignancy in adults, accounting for 80% of all leukemias in patients aged over 60 (Pollyea et al., 2011). It encompasses a cytogenetically heterogeneous group of myeloid malignancies characterized by clonal expansion of abnormally or poorly differentiated cells in the bone marrow, blood and other tissues. In spite of recent progress in diagnosis, stratification and treatment, the disease is still largely incurable [in 60%–65% of patients < 60 years and 85%–95% of patients > 60 years (Dohner et al., 2015)] and overall 5-year survival rates remain poor at only 25% (Ferrara and Schiffer, 2013; Conway O'Brien et al., 2014). Moreover, chemotherapy-induced complete remission of relapsed patients is also not high [10%–25% (Leopold and Willemze, 2002; Greenberg et al., 2004)], while the numbers for secondary AML (s-AML) are even worse, especially in younger patients (Ostgard et al., 2010; Hulegardh et al., 2015). For example, up to 30%–40% of MDS patients develop s-AML with only an 8% 3-year survival rate (Koh et al., 2010). Therefore, additional therapeutic strategies in AML are urgently required and targeting aberrant epigenetics represents a logical approach (Castelli et al., 2018).

Multiple studies have demonstrated a central role for dysregulated epigenetics in AML (The Cancer Genome Atlas Research Network, 2013; Conway O'Brien et al., 2014) and we have previously demonstrated aberrant histone modifications in AML patient samples on chromatin localized to the *RARA* gene (Glasow et al., 2008), which encodes retinoic acid receptor alpha (RARα, NR1B1), a key driver of myeloid differentiation in response to binding of its ligand ATRA (Collins, 2002). One of the histone markers we found to be deregulated was trimethylated lysine 27 on histone H3 (H3K27^me3^), the modification of which may be catalyzed by the EZH2 (KMT6A) protein lysine methyltransferase, an enzymatic subunit of the Polycomb Repressive Complex 2 (PRC2). The importance of the Polycomb-group (PcG) of proteins in embryogenesis and normal hematopoiesis has been highlighted by a number of discoveries showing that aberrations in the functional integrity of PcG complexes can lead to impaired development and maintenance of the hematopoietic system (Lessard et al., 1997, 1998; Ohta et al., 2002; Lessard and Sauvageau, 2003; Park et al., 2003; Kajiume et al., 2004; Kamminga et al., 2006). Regarding EZH2 itself, the nature of its role in cancer and AML in particular appears complex and remains to be fully resolved (Yamaguchi and Hung, 2014; Safaei et al., 2018; Wang and Wang, 2020; Huang et al., 2021), with uncertain implications for this enzyme as a therapeutic target in AML (Eich et al., 2020; Zeng et al., 2022). *EZH2* loss-of-function mutation (Ernst et al., 2010; Kempf et al., 2021) and diminished expression (Gollner et al., 2017) have been linked to AML disease progression and treatment failure. Paradoxically, however, overexpression of EZH2 is also frequently observed in high-risk MDS and AML (Xu et al., 2011; Jiao et al., 2022). The *EZH2* gain-of-function mutation corresponding to Tyr646 that has been described in lymphomas of germinal-center origin has, however, not been identified in AML patients (Ernst et al., 2010). These seemingly contradictory findings may be explained by the finding that EZH2 performs different functions during early and late stages of AML (Basheer et al., 2019).

From the perspective of RARα-mediated myeloid differentiation, EZH2 has been shown to play a role in a rare subtype of AML, acute promyelocytic leukemia (APL), which is generally curable with ATRA-based differentiation therapy (in contrast to non-APL AML) (de The et al., 2017). From a mutational perspective, APL is genetically uncomplex (The Cancer Genome Atlas Research Network, 2013) and characterized by the presence of fusion genes arising from reciprocal translocations involving *RARA*, predominantly with the *PML* gene [t(15;17)(q22;q21), 98% of cases] that subsequently encodes the oncogenic PML-RARα fusion protein. PML-RARα forms homodimers/oligomers independent of RXR, the normal heterodimeric partner of RARα, and does not undergo coregulator exchange leading to transcriptional activation at physiological concentrations of ATRA. The curative action of ATRA (at a therapeutic concentration of 10^−6^ M) in PML-RARα associated APL, as part of a precision medicine combination therapy in conjunction with arsenic trioxide (As_2_O_3_), is predicated on the ability of the compounds to bind directly the RARα and PML moieties of the fusion protein, respectively, to promote its proteasomal degradation and activation of differentiation pathway genes (de The et al., 2017). Evidence points to a lack of efficacy of ATRA-based therapy in non-APL AML as a result, at least in part, of a failure of ATRA to induce proper transcriptional activation of epigenetically repressed genes in the RARα-driven myeloid differentiation pathway (Schenk et al., 2012). EZH2, as part of the PRC2 complex, was found to associate constitutively with PML-RARα, leading to epigenetic deregulation and silencing of PML-RARα target genes (Villa et al., 2007). Furthermore, it was found in two mouse models of AML driven by the MLL-AF9 leukemic fusion gene that *Ezh2* was required for AML progression (Neff et al., 2012) and functioned to inhibit the differentiation program in leukemic stem cells (Tanaka et al., 2012).

The above lines of evidence indicated a complex role for EZH2 in the pathogenesis and treatment of AML but nevertheless one that was potentially therapeutically targetable with precision medicine approaches. Therefore, the aim of this study was to assess whether EZH2 played a role in myeloid differentiation of human non-APL AML, which had not been previously evaluated, and, if so, whether small molecule inhibitors of EZH2 could be effective in combination with ATRA. We tested two structurally and mechanistically distinct small molecules with inhibitory activities against EZH2. Firstly GSK-343, a pyridone-containing, S-adenosyl-L-methionine (SAM) cofactor-competitive EZH2 inhibitor that is 1,000-fold selective over other histone methyltransferases, and 60-fold selective over EZH1 (Verma et al., 2012). We also tested a duel-specificity inhibitor, HKMTI-1-005 (Curry et al., 2015), which is based on BIX-01294, an inhibitor of the closely related G9A (EHMT2) and GLP (EHMT1) H3K9 methyltransferases that primarily mediate dimethylation (H3K9^me2^) (Kubicek et al., 2007). BIX-01294 (a diazepin-quinazolin-amine derivative) is non-competitive with the cofactor SAM, instead competing for binding with the amino acids N-terminal of the substrate lysine residue. EZH2 and G9A/GLP belong to the SET-domain superfamily of protein lysine methyltransferases (Dillon et al., 2005), and based on the ability of G9A to methylate H3K27 in addition to H3K9 (Wu et al., 2011) as well as common features in the recognition motifs for histone binding at repressive sites (Yun et al., 2011), Curry et al reasoned that BIX-01294 could serve as a template for a substrate-competitive dual inhibitor targeting EZH2 and G9A/GLP. While we were initially interested in HKMTI-1-005 as an EZH2 inhibitor, evidence of a role for G9A in AML pathogenesis (Lehnertz et al., 2014) and maintenance of a cancer stem cell phenotype (Haebe et al., 2021) suggested that the dual inhibitory activities of HKMTI-1-005 could be therapeutically advantageous in the context of AML. By taking different approaches to functionally impair EZH2 activity we report here that use of a substrate-competitive dual EZH2-G9A/GLP inhibitor, but not SAM-competitive EZH2 inhibitors can promote differentiation of human AML cells.

## 2 Materials and methods

### 2.1 Cell lines, primary samples and cell culture

HL-60 acute myeloid leukemia (AML FAB M2, Cat# ACC 3) and MV4-11 acute monocytic leukemia (AML FAB M5, Cat# ACC 102) AML cell lines were obtained from the German Collection of Microorganisms and Cell Cultures (DSMZ) and maintained in RPMI 1640 (Cat# R8758, Sigma-Aldrich) supplemented with 10% FBS (Gibco), 100 μg/mL penicillin and 100 μg/mL streptomycin. Bone marrow aspirates of newly diagnosed patients with AML were collected after informed consent and with the approval of the institutional review boards and ethic committees of the University of Jena. Samples were kindly provided by Professor Andreas Hochhaus. Primary cells were cultured in Stemline hematopoietic stem cell expansion medium (Cat# S0189 Sigma-Aldrich).

### 2.2 Reagents, plasmids and virus production

The following reagents were used in this study: Puromycin (Cat# A1113802, Invitrogen); ATRA (Cat# R2625, Sigma-Aldrich); GSK-343 (Cat# 14094, Cayman Chemical); BIX-01294 (Cat# 13124, Cayman Chemical); UNC1999 (Cat# 14621, Cayman Chemical) and Dimethyl sulfoxide (DMSO, Cat# D8418, Sigma-Aldrich). HKMTI-1-005 was provided by Professor Matthew Fuchter. Antibodies used for immunoblot and immunoprecipitation were as follows: rabbit polyclonal anti-H3K27^me3^ (Cat# 07–473, Upstate Biotechnology); rabbit polyclonal anti-H3K9^me2^ (Cat# ab115159, Abcam); rabbit polyclonal anti-GAPDH (Cat# ab9485, Abcam); rabbit polyclonal anti-EZH2 (Cat# ab186006, Abcam); mouse monoclonal anti-EZH2 (clone AC22, Cat# 3147, Cell Signaling Technology); mouse monoclonal anti-RARα (clone 9α-9A6, Cat# 39971, Active Motif); rabbit polyclonal anti-RARα (Cat# sc-773, Santa Cruz). EZH2 shEZH2#1 and shEZH2#2 oligos (reverse/complement of mature antisense sequence GAC​TCT​GAA​TGC​AGT​TGC​T and CAT​GTA​GAC​AGG​TGT​ATG​A respectively) were cloned into pGreenPuro lentiviral expression vectors (System Biosciences) and Z-competent bacteria (Z-competent *E. coli* Transformation Buffer Set, Cat# T3002, Zymo Research) were then transformed with the vectors. Clones were selected and inserts were sequenced (Source BioScience Lifesciences). Plasmid DNA was purified (PureLink HiPure Plasmid Midiprep Kit, Cat# K210004, Invitrogen) and used for transient transfections and the standard production of lentiviruses in HEK-293TN cells (Cat# LV900A-1, System Biosciences). Lentiviral supernatants were prepared, and HL-60 cells infected with viral particles were selected with puromycin and expanded for further experiments.

### 2.3 Cell viability assays

Inhibitors were titrated to determine the concentration values at which 50% of cell viability occurred (GI_50_ values) in each cell line. In brief, cells were seeded (∼20,000 cells per well) in 96-well flat bottom plates in a total of 150 µL culture media ± compounds and grown for 72 h in triplicates. Cell viability was determined using the CellTiter-Glo Luminescent Cell Viability Assay (Cat# G7570, Promega).

### 2.4 Immunoblot analysis

Cells were lysed in RIPA buffer (Cat# R0278, Sigma) for immunoblot analysis. Protein concentration from whole cell lysate was measured (DC Protein Assay kit II, cat# 5000112, Bio-Rad) and 10 µg–20 µg of protein per well was separated on 10% NextGel (Cat# M256, Amresco) or gradient pre-cast Mini-PROTEAN TGX gels (BioRad). Proteins were transferred on Hybond ECL membrane (GE Healthcare). Membranes were blocked using 5% milk or BSA in PBS-Tween (0.1%), incubated overnight at 4°C with primary antibodies (described above), washed in PBS-Tween and incubated with anti-mouse (Cat# 155–035-174) and anti-rabbit (Cat# 211–032-171) light chain specific antibodies HRP-secondary antibodies (Jackson ImmunoResearch). Bands were visualized using ECL kit (Thermo Scientific) and X-ray films (Kodak) or Gel Doc system (BioRad).

### 2.5 Flow cytometry

FACS analysis of CD11b (MAC-1) expression by AML cell lines and apoptosis analysis of patient samples after drug treatment were performed using an APC-conjugated human CD11b-specific mouse monoclonal antibody at a 1:10 dilution (Cat# 555388, BD Pharmingen) on a BD LSRII FACS machine (Becton Dickinson) with FACS Diva software. For analysis of apoptosis, the Annexin-V FITC apoptosis detection kit II (Cat# 556570, BD Pharmingen) was used according to the manufacturer’s instructions with Propidium Iodide (Cat# R37169, Invitrogen) at 1 μg/mL on Guava EasyCyte flow cytometer with InCyte software (Merck).

### 2.6 Giemsa staining

For the morphological analysis of myeloid cell differentiation, cytospins were prepared by centrifugation in 150 µL PBS at a speed of 300 rpm for 5 min using Superfrost Plus (VWR) positively charged glass slides. Cytospun slides were stained at room temperature with May-Grünwald-Giemsa (Sigma) and cellular morphology was examined using an Axioscope 2 plus microscope with an AxioCam MRc camera (Zeiss).

### 2.7 Duolink proximity ligation assay

Duolink *in situ* proximity ligation assays (PLA, Sigma Aldrich) were performed on HL-60 AML cells fixed in 4% paraformaldehyde for 20 min, permeabilized with 0.5% Triton X-100, and blocked with 1% BSA for 30 min at room temperature followed by incubation with paired primary antibodies, anti-EZH2 and anti-RARα, overnight at 4°C. PLA detection was performed as recommended by the manufacturer. Images were taken and analyzed using the Zeiss LSM700 confocal microscope and analyzed using Duolink image analysis software.

### 2.8 Statistics

Experiments were performed at least 3 times, unless otherwise indicated. Error bars represent mean ± standard deviation. Statistical analysis was performed using the unpaired *t*-test unless otherwise indicated. *p*-values < 0.05 were considered as statistically significant. Computations were performed using Prism (GraphPad version 9.5.0, San Diego, CA).

### 2.9 Expression microarray analysis

GeneChip Human Gene 1.0 ST Array and GeneChip, WT PLUS Reagent Kit, GeneChip WT Terminal Labeling and Controls Kit, GeneChip Hybridization, Wash, and Stain Kit, processed with GeneChip Fluidics Station 450, GeneChip Hybridization Oven 645 and GeneChip Scanner 3,000, were used and standard protocols provided by Affymetrix and Ambion were followed. In brief, total RNA was extracted from 72 h-treated cells and control cells, cDNA, cRNA and then single-stranded cDNA were generated with amplification and purification steps in between, the latter being digested, labelled and hybridized to the oligonucleotide probes on the chips overnight. Staining, washing and scanning on the final day was carried out and the images were processed by the Affymetrix GeneChip Operating Software (GCOS) to produce .CEL files, which were then used for the bioinformatics analysis of the arrays.

Arrays were normalized with Partek Genomics Suite (https://www.partek.com/partek-genomics-suite/) using GC-RMA, after which “batch-remove” function in Partek was applied to normalize for batch-to-batch and date-to-date variation between samples. False discovery rate (FDR) testing across all conditions was applied to generate gene lists with statistically significant (FDR ≤ 0.05) differentially expressed genes between the conditions. The resulting gene lists were further refined by setting fold change (1.3-fold change) and overall expression (top 40th percentile) cut-offs and were analyzed by the following online tools: Database for Annotation, Visualization and Integrated Discovery (DAVID, https://david.ncifcrf.gov) and Gene Set Enrichment Analysis (GSEA, https://www.gsea-msigdb.org). For GSEA, .gct and .cls files were manually created and uploaded in the software. *q*-values (equivalent to FDR-adjusted p-values) ≤ 0.05 were set as a cut-off and enriched categories were then further analyzed using online tools including Venny (http://bioinfogp.cnb.csic.es/tools/venny/index.html).

## 3 Results

### 3.1 Dual inhibition of G9A and EZH2 promotes myeloid differentiation and cell death in AML cells

**FIGURE 1:**
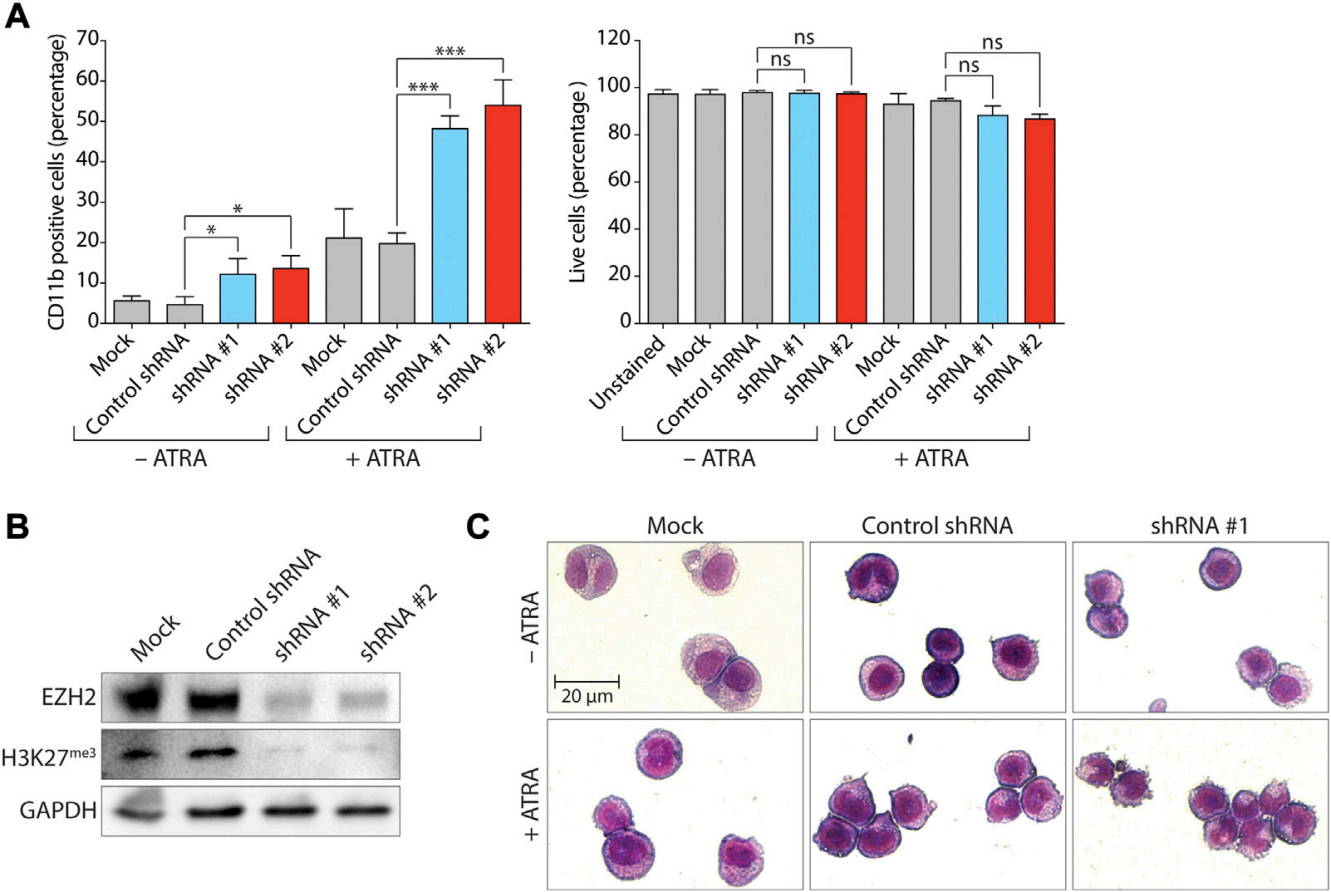
Knockdown of *EZH2* promotes differentiation of HL-60 AML cells by ATRA. **(A)** Expression of the myeloid differentiation marker CD11b (left panel), and proportion of live cells (PI-negative population, right panel) following lentivirally-mediated knockdown (KD) of *EZH2* expression and analysis by flow cytometry. *EZH2* KD was achieved using two different short-hairpin RNA sequences (shRNA #1 and #2) as indicated. A non-targeting shRNA was used as a negative control. Values represent the means of three experiments and error bars denote standard deviations. *p < 0.05; ***p < 0.001; ns, no statistical significance. **(B)** Immunoblot analysis of levels of EZH2 protein and trimethylated H3 Lys27 (H3K27^me3^) following *EZH2* KD. Transduced cells were selected with puromycin and expanded for a minimum of 10 days. GAPDH was used as a loading control. **(C)** Cell morphology of cells analyzed by May-Grunwald Giemsa staining following *EZH2* KD (shRNA #1) +/− ATRA (0.1 µM for 72 h).

In order to assess the impact of EZH2 expression on myeloid differentiation of human non-APL AML, we first examined the effect of *EZH2* knockdown (KD) via lentiviral transduction of two short hairpin sequences (shEZH2#1 and shEZH2#2) targeting separate sequences on *EZH2* mRNA in *MYC*-amplified human HL-60 cells (AML FAB M2) (Collins et al., 1977), a long-established model system in this context (Birnie, 1988). HL-60 cells do not have any reported mutations for *EZH1/KTM6B*, *EZH2/KTM6A*, *G9A*/*EHMT2* or *GLP*/*EHMT1* as reported by the Catalogue of Somatic Mutations in Cancer (COSMIC v97, https://cancer.sanger.ac.uk/cosmic) and cBioPortal (https://www.cbioportal.org/) analysis of the Cancer Cell Line Encyclopedia (https://sites.broadinstitute.org/ccle/) (Cerami et al., 2012; Ghandi et al., 2019). According to available data on cBioPortal, mutation of *EZH2* is uncommon in AML cell lines, with a single cell line identified as harboring a Y646C predicted gain-of-function mutation (SKM-1) (Palau et al., 2017) and three cell lines harboring predicted loss-of-function mutations: PL-21 (Quentmeier et al., 2003), OCI-AML5 (Wang et al., 1991), and P31/FUJ (Hirose et al., 1982). Treatment with 1 µM ATRA typically differentiates HL-60 cells effectively towards granulocyte-like cells, with more than 90% of cells reflecting band (immature) or segmented (mature) neutrophils (Manda-Handzlik et al., 2018). Upon ATRA treatment, these cells express the myeloid lineage cell-surface CD11b marker but not CD14 that is associated with maturation to monocytes and macrophages. In order to evaluate the impact of compounds on the promotion of myeloid differentiation of HL-60 cells, experiments were performed using a sub-optimal ATRA concentration of 0.1 µM. Consistent with results indicating a role for EZH2 in blocking differentiation in an MLL-AF9 driven mouse model of AML (Tanaka et al., 2012), *EZH2* KD induced differentiation in HL-60 cells as measured by flow cytometric analysis of the percentages of cells expressing CD11b (CD11b positive, CD11b+ cells) in either the absence or presence of ATRA (ranging between 2.44-fold and 3-fold, Figure 1A, left panel; Supplementary Figure S1). *EZH2* KD was confirmed by immunoblot analysis of EZH2 protein and H3K27^me3^ (Figure 1B) and in agreement with the increase in the percentage of CD11b+ cells. After May-Grünwald-Giemsa staining, increased numbers of cells displaying morphological changes consistent with myeloid differentiation (nuclear lobulation and increased cytoplasmic:nuclear ratio) were observed when *EZH2* KD cells treated with ATRA were compared with cells treated with ATRA alone (Figure 1C). The increase in expression of CD11b in *EZH2* KD cells treated with ATRA was not accompanied by an increase in cells undergoing necrosis or late-stage apoptosis as measured by propidium iodide (PI) uptake (Figure 1A, right panel), but analysis of cell morphology showed the presence of apoptotic blebs on the cell surface that was indicative that the cells were undergoing apoptosis (Figure 1C). It has been demonstrated previously that apoptotic blebs can form prior to a loss of overall membrane permeability (Wickman et al., 2013).

**FIGURE 2:**
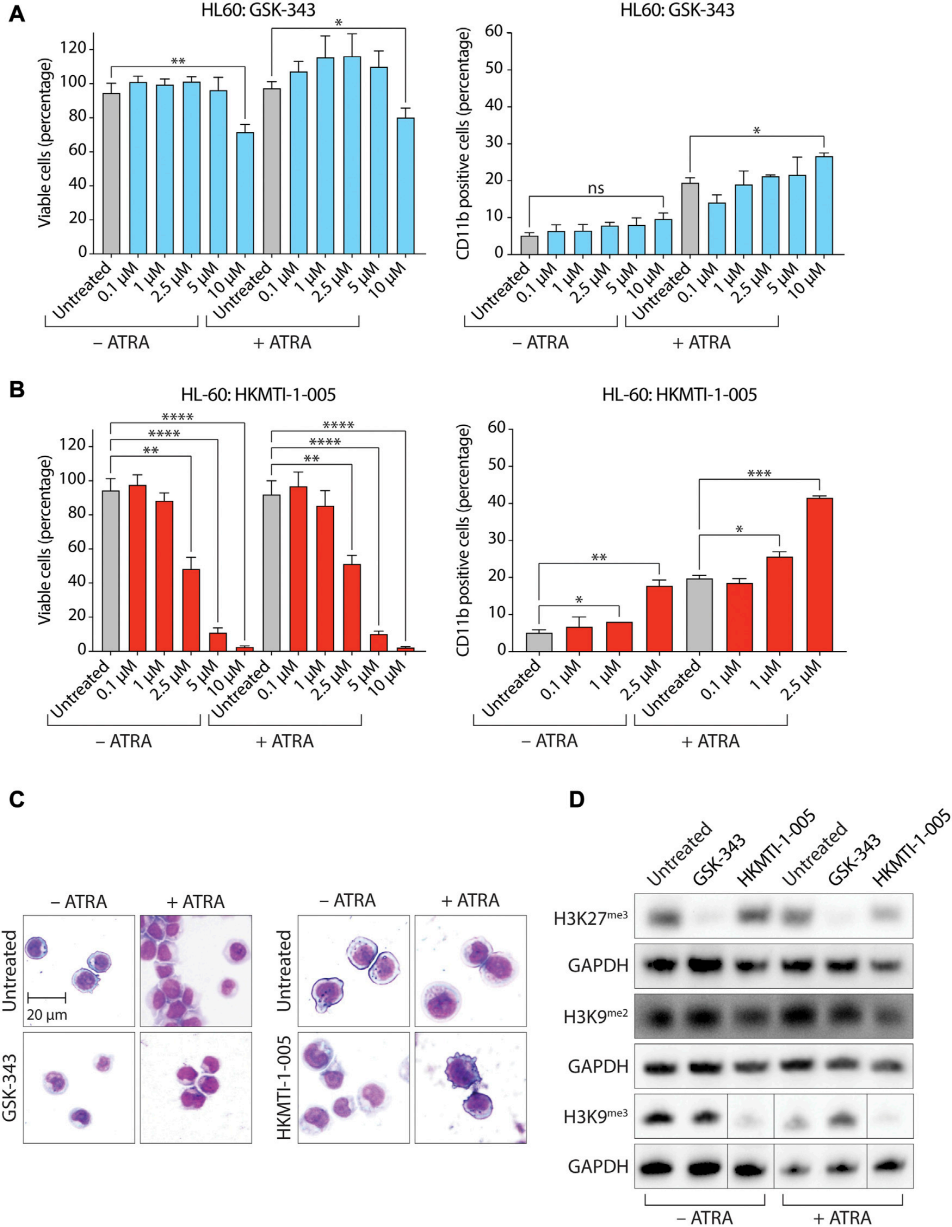
Dual inhibition of EZH2-G9A/GLP by HKMTI-1-005 promotes differentiation of HL-60 AML cells by ATRA. **(A)** Proportion of viable cells as determined by CellTiter-Glo cell viability assay (left panel), and expression of the myeloid differentiation marker CD11b (right panel) following treatment with the indicated concentrations of GSK-343 +/− 0.1 µM ATRA for 72 h. **(B)** Proportion of viable cells as determined by CellTiter-Glo cell viability assay (left panel) and expression of the myeloid differentiation marker CD11b (right panel) following treatment with the indicated concentrations of HKMTI-1-005 +/− 0.1 µM ATRA for 72 h. Values represent the means of three experiments (viability assays) or two experiments (CD11b flow cytometry). Error bars denote standard deviations. *p < 0.05; **p < 0.01; ***p < 0.001; ****p < 0.0001; ns, no statistical significance. **(C)** Cell morphology of cells analyzed by May-Grunwald Giemsa staining following treatment with 10 µM GSK-343 (left panel) or 2.5 µM HKMTI-1-005 (right panel) for 72 h. Treatments were performed +/− 0.1 µM ATRA. **(D)** Immunoblot analysis of levels of trimethylated H3 Lys27 (H3K27^me3^), dimethylated H3 Lys9 (H3K9^me2^) and trimethylated H3 Lys9 (H3K9^me3^) following treatment (72 h) with 10 µM GSK-343 or 2.5 µM HKMTI-1-005 +/− 0.1 µM ATRA as indicated. GAPDH was used as a loading control.

We next tested GSK-343 in HL-60 (Figure 2; Supplementary Figures S2, S3) and MV4-11 cells (Lange et al., 1987), which were established from a patient with biphenotypic B-myelomonocytic leukemia (AML FAB M5) (Supplementary Figures S2, S4, S5). MV4-11 cells harbor a t(4;11)(q21;q23) *MLL*/*AF4* (*KMT2A*/*AFF1*) chromosome translocation and a *FLT3* internal tandem duplication (Quentmeier et al., 2003). MV4-11 cells do not have any reported mutations for *EZH1/KTM6B*, *EZH2/KTM6A* or *G9A*/*EHMT2*, but do harbor a missense mutation in *G9A*/*EHMT1* resulting in a conservative leucine to valine substitution at amino acid position 1059 (L1059V) (Cerami et al., 2012; Ghandi et al., 2019). When treated with ATRA, MV4-11 cells undergo myelomonocytic differentiation, expressing both CD11b and CD14 markers (Gocek et al., 2012; Ma et al., 2016). As with HL-60 cells, experiments using MV4-11 were performed using a sub-optimal ATRA concentration of 0.1 µM. We found that GSK-343 moderately decreased the percentage of metabolically active, viable, cells as measured by ATP quantitation with CellTiter-Glo for both HL-60 (Figure 2A, left panel) and MV4-11 cells (Supplementary Figure S4A, left panel) with GI_50_ values of 13.22 µM (Supplementary Figure S2A) and 10.12 µM (Supplementary Figure S2C), respectively. We did not observe the presence of apoptotic blebs on the cell surface of HL-60 cells (Figure 2C, left panel), but blebbing was evident on MV4-11 in either the absence or presence of ATRA (Supplementary Figure S4C, left panel). For HL-60 cells, while GSK-343 treatment did not induce myeloid differentiation in the absence of ATRA, it caused a small but significant increase in the percentage of CD11b+ cells in the presence of ATRA (Figure 2A, right panel and Supplementary Figure S3), although this was not accompanied by a clear change in cell morphology associated with myeloid differentiation (Figure 2C, left panel). GSK-343 treatment failed to induce myeloid differentiation in MV4-11 cells as evidenced by a lack of increase in the proportion of CD11b+ cells or changes in cell morphology (Supplementary Figures S4A, right panel; (Supplementary Figures S4C, left panel and Supplementary Figure S5). We also tested the pyridone-containing and SAM-competitive EZH1 and EZH2 inhibitor UNC1999 (Konze et al., 2013) for its ability to induce cell death and myeloid differentiation in HL-60 cells and the results obtained were similar to those for GSK-343 (Supplementary Figure S6). UNC1999 decreased cell viability in HL-60 cells in a dose-dependent manner with a GI_50_ value of 7.22 µM (Supplementary Figure S6A,B). UNC1999 did not induce myeloid differentiation in the absence of ATRA but, consistent with GSK-343, did cause a small but significant increase in the percentage of CD11b+ cells in the presence of ATRA (Supplementary Figure S6C).

Inhibition of EZH2 with HKMTI-1-005 decreased cell viability for both HL-60 (Figure 2B, left panel) and MV4-11 cells (Supplementary Figure S4B, left panel) in a dose-dependent manner at lower concentrations than GSK-343, with GI_50_ values of 2.43 µM (Supplementary Figure S2B) and 2.45 µM (Supplementary Figure S2C), respectively. Apoptotic blebbing was evident on both HL-60 (Figure 2C, right panel) and MV4-11 (Supplementary Figure S4C, right panel) cells treated with HKMTI-1-005 in the presence of ATRA. In contrast with GSK-343, HKMTI-1-005 treatment increased the percentage of CD11b+ HL-60 cells both in the absence (3.2-fold) and presence (2.1-fold) of ATRA (Figure 2B, right panel and Supplementary Figure S3), and promoted a cell morphology associated with myeloid differentiation Figure 2C, right panel). Similarly, HKMTI-1-005 treatment increased the percentage of CD11b+ MV4-11 cells 4.2-fold and 2.9-fold in the absence and presence of ATRA, respectively, (Supplementary Figure S4B, right panel and Supplementary Figure S5), also promoting a cell morphology associated with myeloid differentiation (Supplementary Figure S4C, right panel).

**FIGURE 3:**
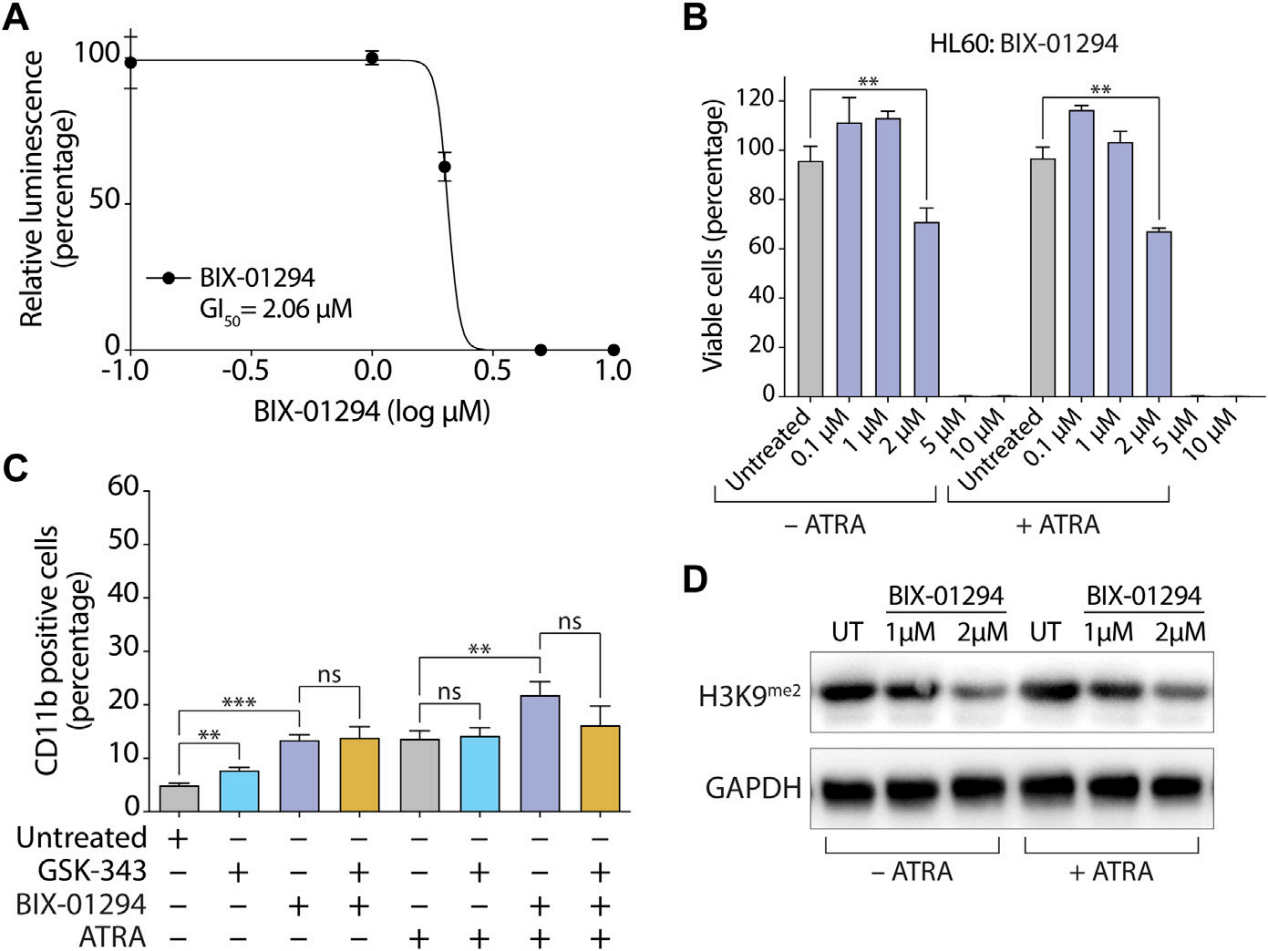
Inhibition of G9A/GLP by BIX-01294 promotes moderate differentiation in HL-60 cells. **(A)** Determination of GI_50_ concentration for BIX-01294 in HL-60 cells. Cells were seeded at 20,000 cells per well and treated for 72 h with different concentrations of BIX-01294 as indicated. The growth inhibitory (GI_50_) concentration was determined by measuring cell viability with CellTiter-Glo and generating a dose-response curve (GraphPad Prism, version 9.5.0). **(B)** Proportion of viable cells as determined by CellTiter-Glo cell viability assay following treatment with the indicated concentrations of BIX-01294 +/− 0.1 µM ATRA for 72 h. **(C)** Flow cytometry quantification of the percentage of CD11b positive cells after treatment with 2 µM BIX-01294 and/or 10 µM GSK-343 +/− 0.1 µM ATRA for 72 h. Values represent the means of three experiments and error bars denote standard deviations. **p < 0.01; ***p < 0.001; ns, no statistical significance. **(D)** Immunoblot analysis of levels of dimethylated H3 Lys9 (H3K9^me2^) following treatment with 2 µM BIX-01294 +/− 0.1 µM ATRA for 72 h. GAPDH was used as a loading control.

As expected, treatment with 10 µM GSK-343 almost completely abolished H3K27^me3^ levels in both HL-60 (Figure 2D) and MV4-11 (Supplementary Figure S4D) cells. However, treatment with the GI_50_ concentration of HKMTI-1-005 (2.5 µM) diminished, rather than abolished, H3K27^me3^ levels in the presence of ATRA in both HL-60 (Figure 2D) and MV4-11 (Supplementary Figure S4D) cells. A decrease but not abolition of H3K27^me3^ levels with 2.5 µM HKMTI-1-005 in the AML cell lines examined here is in agreement with previous data for MDA-MB-231 breast cancer cells, where treatment with an approximate IC_50_ concentration of HKMTI-1-005 diminished H3K27^me3^ by around half (Curry et al., 2015). Consistent with its G9A/GLP inhibitory activity, 2.5 µM HKMTI-1-005 treatment also reduced levels of H3K9^me2^ and H3K9^me3^ both in the absence and presence of ATRA (Figure 2D). To evaluate the effect of G9A/GLP inhibition alone on cell viability and myeloid differentiation, we also treated HL-60 cells with BIX-01294. In agreement with previous results (Savickiene et al., 2014), we found that G9A/GLP inhibition with BIX-01294 at the GI_50_ concentration of 2 µM (higher concentrations were extremely cytotoxic, Figures 3A, B) also promoted a moderate increase in the percentage of CD11b+ HL-60 cells (Figure 3C), which was accompanied by a reduction in H3K9^me2^ (Figure 3D). These results are consistent with a study using another substrate-competitive G9A/GLP inhibitor, A-366, which was found to increase CD11b expression and decrease cell viability with concomitant diminution in H3K9^me2^ levels (Pappano et al., 2015). Interestingly, we found that co-treatment with BIX-01294 and GSK-343 did not increase the proportion of CD11b+ cells in the absence of ATRA and led to a small decrease in the proportion of CD11b+ cells in the presence of ATRA (Figure 3C).

**FIGURE 4:**
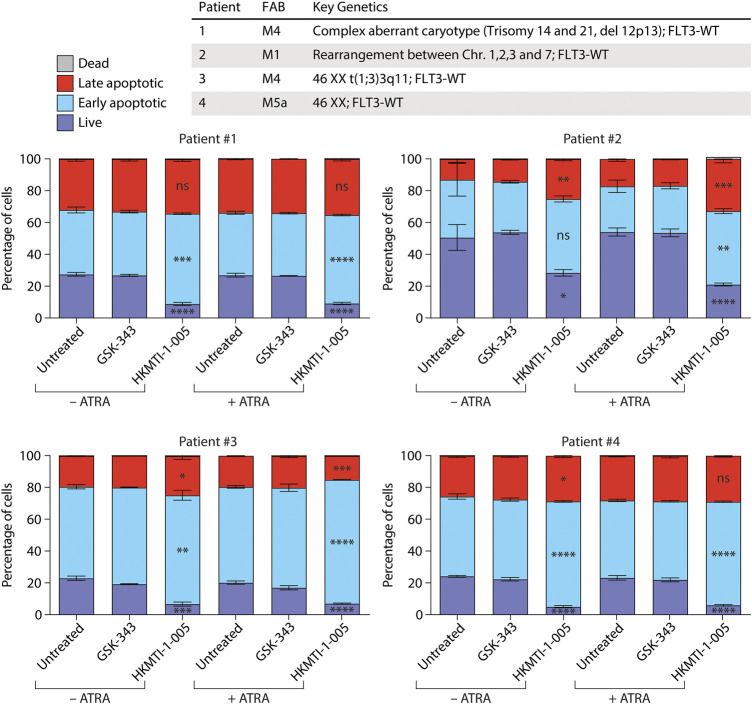
HKMTI-1-005 diminishes cell viability of primary AML cells. Primary AML cells from patients were treated with 10 µM GSK-343 or 2.5 µM HKMTI-1-005 for 48 h. Treatments were performed +/− 0.1 µM ATRA. The proportions of live, early apoptotic, late apoptotic and dead cells were evaluated by uptake of propidium iodide (PI) and Annexin V as measured by flow cytometry. Values represent the means of three experiments and error bars denote standard deviations. *p* values refer to percentages of live, early or late apoptotic cells treated with HKMTI-1-005 compared with untreated, or HKMTI-1-005 plus ATRA compared with ATRA alone. *p < 0.05; **p < 0.01; ***p < 0.001; ****p < 0.0001; ns, no statistical significance. No statistically significant change was observed in the percentages of live, early or late apoptotic cells for cells treated with GSK-343 compared with untreated, or GSK-343 plus ATRA compared with ATRA alone. No statistically significant change was observed in dead cells.

Lastly, we tested GSK-343 and HKMTI-1-005 against four samples of primary AML cells (Figure 4). We found that treatment with the HKMTI-1-005 concentration used for the cell lines for 48 h −/+ ATRA effectively diminished the proportion of live primary AML cells, with a concomitant increase in early apoptotic cells. There was no increase in the proportion of dead cells at the 48-h time point, and two samples, Patient #2 and Patient #3, showed an increase in the percentage of late apoptotic cells. Treatment with 10 µM GSK-343 −/+ ATRA or ATRA alone, by contrast, did not affect the proportion of live, early or late apoptotic primary AML cells. We failed to see induction of CD11b expression for any of the four AML patient samples tested here (data not shown). It should be noted that we and others have observed in ATRA-based therapy a high level of variation in the induction of cell-surface CD11b, a lack of correlation between decreases in cell viability and CD11b induction, and also that morphological features associated with granulocytic differentiation do not necessarily correlate with the presence CD11b (Schenk et al., 2012; Klobuch et al., 2018; Kahl et al., 2019). The limited number of samples studied here, as well as the 48-h treatment (which was used due to non-specific decrease in cell viability at 72 h), mean that further investigation is required as for this study we had limited access to primary samples.

### 3.2 *EZH2* KD and HKMTI-1-005 treatment, but not SAM-competitive EZH2 inhibition with GSK-343, elicit transcriptional signatures associated with myeloid differentiation

**FIGURE 5:**
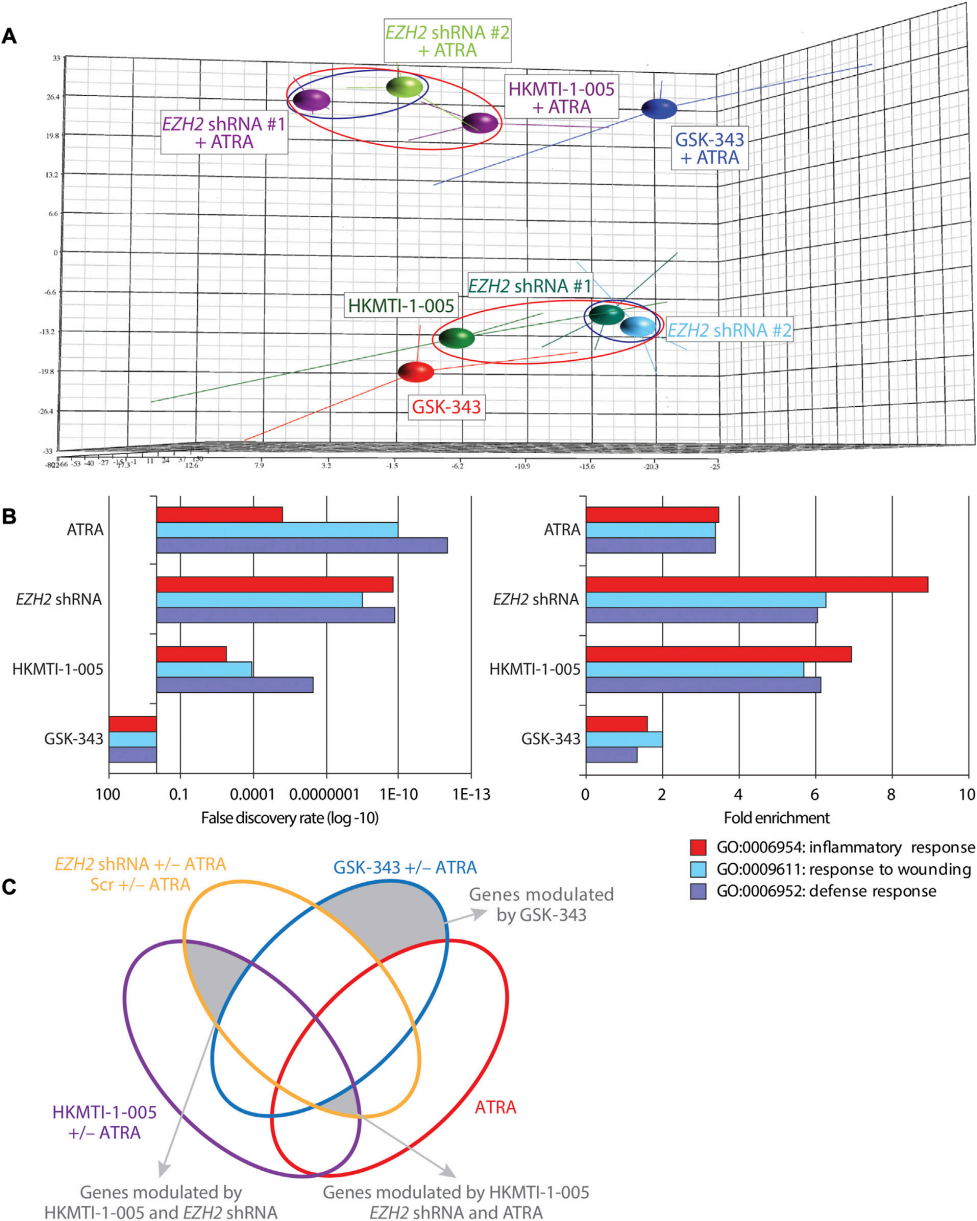
Differential gene expression following *EZH2* knockdown or inhibition with GSK-343 or HKMTI-1-005 in HL-60 AML cells. **(A)** Principal component analysis (PCA; covariance) of GCRMA-normalized expression array samples in biological triplicates represented with centroids as annotated: two different *EZH2*-targeting shRNAs and one non-targeting control (days ∼10–20 post transduction), 10 µM GSK-343 or 2.5 µM HKMTI-1-005 +/− 0.1 µM ATRA following treatment for 72 h. Red or blue ellipses highlight specific grouping of samples of interest. **(B)** Gene ontology (GO) analysis of upregulated genes following *EZH2* KD or drug treatment as annotated (0.1 µM ATRA, 10 µM GSK-343, 2.5 µM HKMTI-1-005 for 72 h) showing enrichment score (left panel) and fold-enrichment over control (right panel) of genes belonging to the indicated categories. **(C)** Venn diagram showing groups of genes taken for further analysis that belong exclusively to GSK-343 treated cells, or are shared only between *EZH2* KD and HKMTI-1-005 treated cells, or between *EZH2* KD, HKMTI-1-005 and ATRA, but not GSK-343 treated cells. Gene groups are shaded grey and indicated.

We next investigated the consequences for transcriptional programs in HL-60 cells as a result of inhibiting the activities of EZH2 by *EZH2* KD, or treatment with GSK-343 or HKMTI-1-005, either alone or in combination with ATRA. Following 72 h treatment, we performed gene expression arrays and transcriptomic analysis. Initial post-normalization principal component analysis (PCA) revealed that, consistent with its role as a ligand for RARα (a key mediator of myeloid differentiation) (Collins, 2002), ATRA treatment caused the greatest change to the transcriptomic program in HL-60 cells. However, while the transcriptional signatures for HL-60 cells following single agent GSK-343 and HKMTI-1-005 treatment were more similar, HKMTI-1-005/ATRA co-treatment resulted in a transcriptional signature that resembled that for ATRA-treated *EZH2* KD cells as compared with GSK-343/ATRA co-treatment (Figure 5A). In accordance with the minimal differentiation response in HL-60 cells treated with GSK-343, the transcriptional signature of these cells was more similar to that of untreated control as evidenced by the smaller variation along the *z*-axis (PC2) between these two samples compared to that between HKMTI-1-005 treated cells and untreated control, especially in the presence of ATRA (Supplementary Figure S7A). Thus, PCA analysis indicated that SAM-competitive inhibition of EZH2 failed to trigger a transcriptional program associated with myeloid differentiation, regardless of the ability of GSK-343 to effectively deplete global H3K27^me3^ levels as measured by immunoblot analysis. Gene ontology (GO) analysis of upregulated genes (FDR < 0.05, FC = ±1.3) provided further support for the finding that *EZH2* KD and EZH2 targeting via HKMTI-1-005 induced similar transcriptomic changes that are likely to underpin the previously observed phenotypes of myeloid differentiation, while GSK-343 failed to direct the fate of the cells towards a more mature state. Unsurprisingly, the presence of ATRA was insufficient to activate certain expression signatures related to myeloid differentiation (Supplementary Figure S7B), and we found that in the absence of ATRA *EZH2* KD and inhibition with HKMTI-1-005, but not SAM-competitive inhibition with GSK-343, upregulated the same ATRA-driven transcriptional programs that are characteristic for mature myeloid cells including those involved in inflammatory response, response to wounding, and defense response (Figure 5B).

**FIGURE 6:**
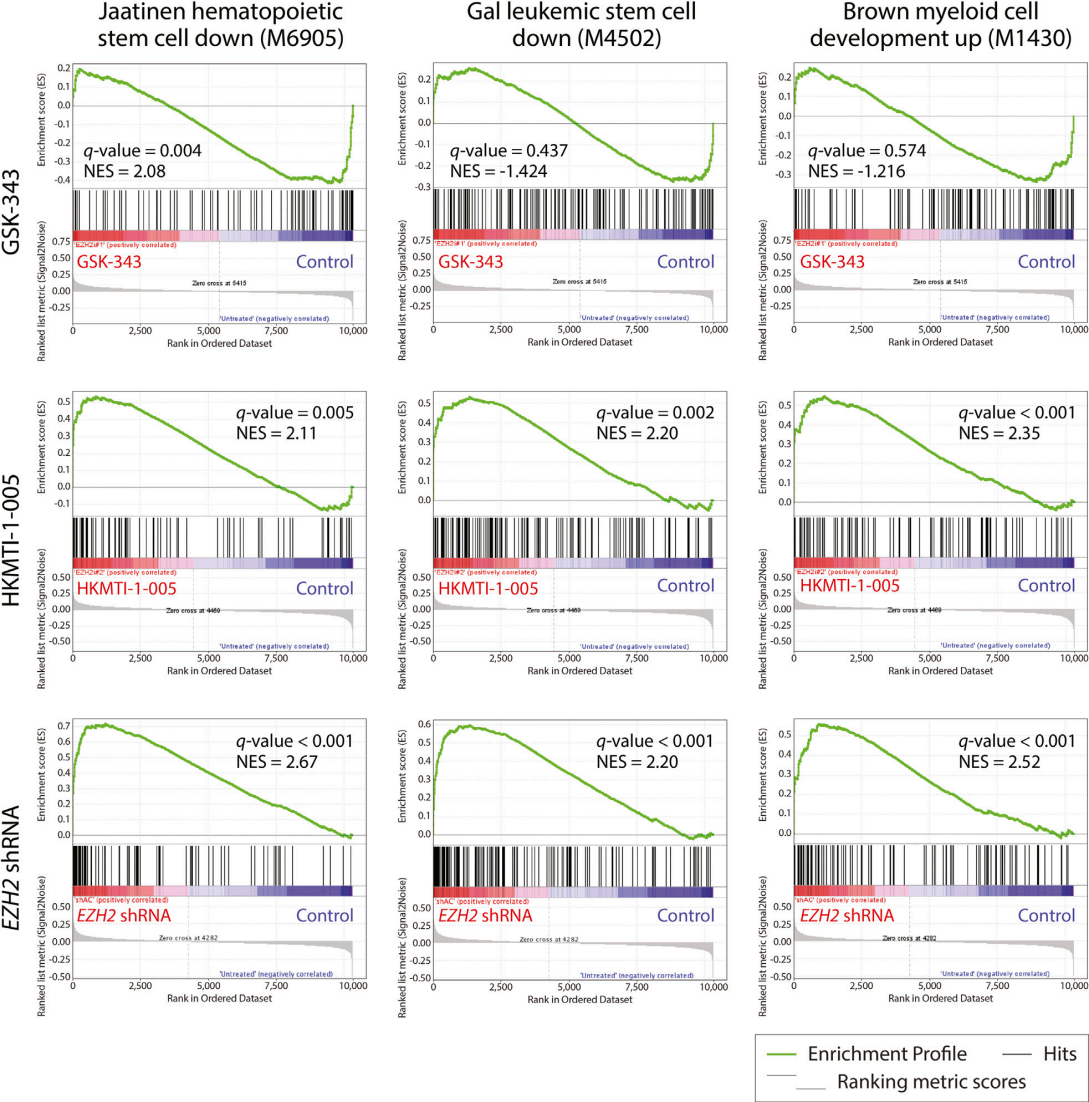
Gene set enrichment analysis (GSEA) of gene expression in HL-60 cells reveals opposite effects of GSK-343 and HKMTI-1-005 on stem cell and differentiation programs. Gene expression data were generated by expression microarray analysis following 72 h treatment with 10 µM GSK-343 or 2.5 µM HKMTI-1-005, or *EZH2* KD *versus* untreated control and analyzed using GSEA to extract biological knowledge. Highly significantly enriched gene sets are indicated by their standard and systematic names. The most upregulated genes in treatment or *EZH2* KD samples are shown on the left side (red), while the most upregulated genes in the control are shown on the right side (blue). Black bars represent the positions of the treatment or *EZH2* KD samples *versus* vehicle control upregulated signature genes in the ranked list. Green curves represent the evolution of gene density. Normalized enrichment scores (NES) reflect the degree to which genes were overrepresented. When the distribution is random, the enrichment score is zero. Enrichment of signature genes at the top of the ranked list results in a large positive deviation of the NES from zero. *q*-value = FDR-adjusted *p*-value.

We next examined all the differentially expressed genes used for the GO analysis (Figure 5C) to attempt to address the differences and similarities between the three ways of targeting EZH2 and gain more insight into the enhanced differentiation or lack of such (Supplementary Tables S1, S2). We found that genes exclusively upregulated by GSK-343 are connected to oncogenic/stem cell programs (e.g., WNT signaling), and included genes involved in transcriptional control, of which *GATA2* (Nandakumar et al., 2015), *LEF1* (Petropoulos et al., 2008), *SMYD2* (Sakamoto et al., 2014) and *MLLT3* (AF9) have been strongly implicated in leukemogenesis. On the other hand, *EZH2* KD and HKMTI-1-005 treatment, both in the presence and absence of ATRA, confirmed the myeloid maturation of the cells through upregulation of the expression of genes encoding cell surface receptors that are functionally important including *CD36* and *ITGAM* (CD11b). Among the genes that were downregulated, we found that GSK-343 treatment not only failed to trigger myeloid maturation, but also downregulated the expression of receptors that are implicated in myeloid function, including *SLAMF7*, *GPR65* and *FCER2* (CD23) (Lattin et al., 2007; Mossalayi et al., 2009; Beyer et al., 2012). Lastly, we carried out gene set enrichment analysis (GSEA) in order to understand the transcriptional changes that characterize the differentiation phenotype observed following *EZH2* KD and HKMTI-1-005 treatment. We found that targeting of EZH2, via gene knockdown or substrate-competitive inhibition, affected the “stemness” transcriptional signatures of the cells. We observed downregulation of hematopoietic and leukemic stem cell attributes, including the gene sets “Jaatinen hematopoietic stem cell down” (M6905), and “Gal leukemic stem cell down” (M4502), and enhancement of transcriptional programs that are involved in myeloid cell development (Figure 6), including the gene set “Brown myeloid development up” (M1430). In contrast, GSK-343 induced the opposite effect. There was upregulation of hematopoietic stem cell signatures and a concomitant failure to activate downregulation of leukemic stem cell attributes and upregulation of myeloid differentiation programs (negative enrichment scores). This strong transcriptional basis confirmed the inability of GSK-343 inhibition of EZH2 to trigger differentiation.

### 3.3 EZH2 associates with RARα following ATRA treatment and HKMTI-1-005, but not GSK-343, enhances this association

**FIGURE 7:**
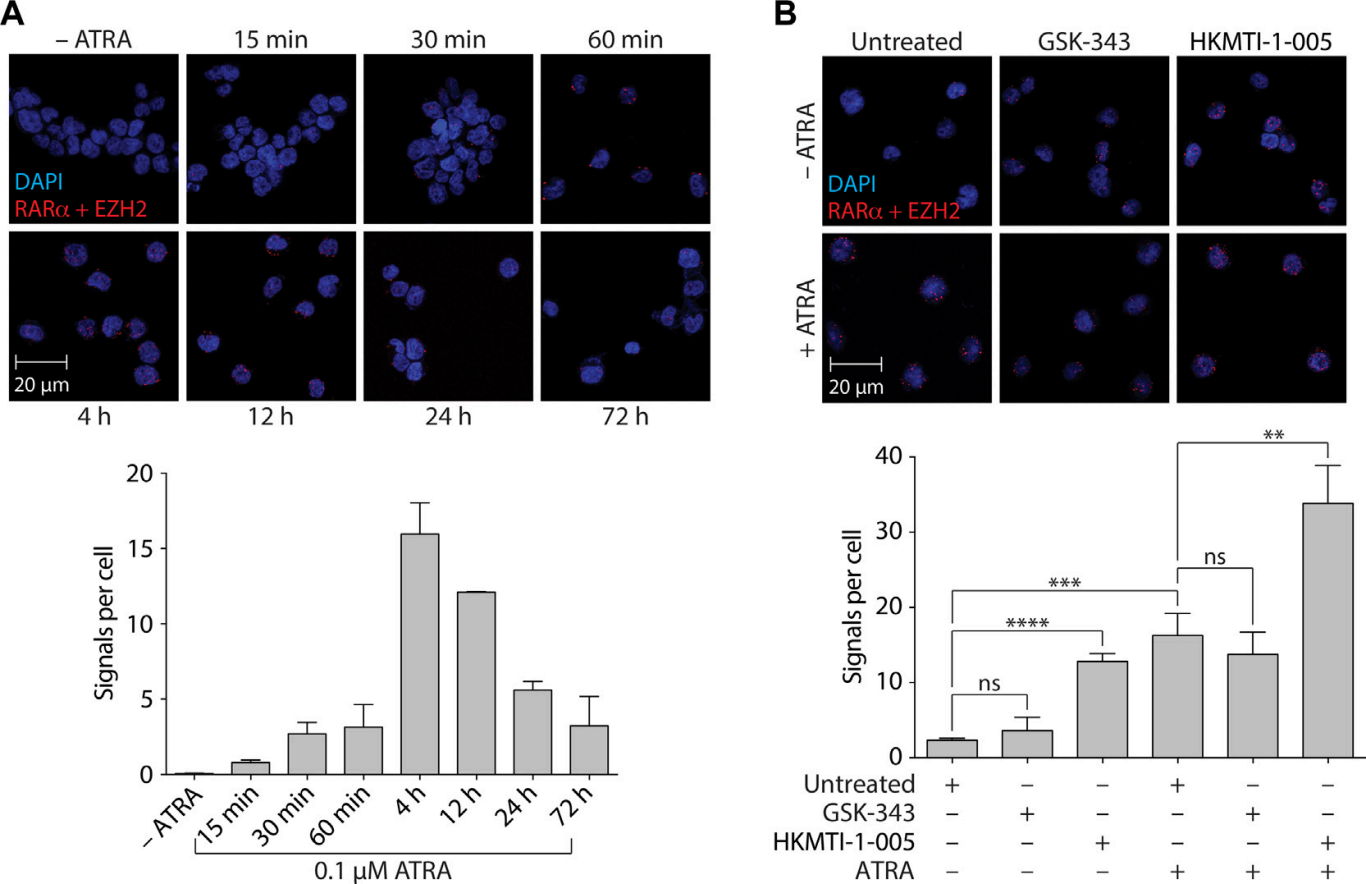
ATRA-mediated differentiation promotes an association between RARα and EZH2 in HL-60 cells that is inhibited by GSK-343 and enhanced by HKMTI-1-005. **(A)** Proximity ligation assay (PLA) analyzing RARα/EZH2 complexes following 0.1 µM ATRA treatment for the indicated times. **(B)** PLA analyzing RARα/EZH2 complexes following treatment with 10 µM GSK-343 or 2.5 µM HKMTI-1-005 for 4 h. Treatments were performed +/− 0.1 µM ATRA as indicated. Upper panels show representative fields of view of cells indicating proximity (< 40 nm) of antibody conjugated PLA probes that have been ligated, amplified, and detected with complementary fluorescent probes. Red dots represent the presence of RARα/EZH2 interactions. Scale bars, 20 µm. Cell nuclei are counterstained with DAPI (blue). Lower panels show mean values of signals (red dots) per cell representing RARα/EZH2 interactions. Values represent the means of two experiments **(A)** or three experiments **(B)**, and error bars denote standard deviations. **p < 0.01; ***p < 0.001; ****p < 0.0001; ns, no statistical significance.

Our results indicated that inhibition of canonical EZH2 activity (i.e., methylation of H3K27) via pyridone-containing, SAM-competitive inhibitors such as GSK-343 and UNC1999 was not effective in terms of promoting ATRA-based myeloid differentiation. We therefore sought to identify whether treatment with GSK-343 or HKMTI-1-005 could differentially impact non-canonical EZH2 activity in the context of RARα-mediated myeloid differentiation given that EZH2 had previously been shown to interact with RARα (albeit with less affinity as compared with PML-RARα) (Villa et al., 2007). Using the proximity ligation assay (PLA) (Fredriksson et al., 2002), we first performed a time course experiment with ATRA and found that upon treatment there was a rapid increase in the number of signals per cell, indicative of increased RARα/EZH2 interactions, which peaked between 4 h and 8 h post-treatment (Figure 7A). We next assessed the impact of GSK-343 or HKMTI-1-005 treatment on increased RARα/EZH2 interactions at the 4-h timepoint. The addition of GSK-343 did not significantly increase the number of signals per cell as measured by PLA in the absence of ATRA and led to a small but non-significant decrease in the number of signals per cell following ATRA treatment (Figure 7B). The addition of HKMTI-1-005, by contrast, significantly increased the number of signals per cell as measured by PLA both in the absence and presence of ATRA (5.5-fold and 2.1-fold, respectively) (Figure 7B).

## 4 Discussion

In the now more than 60 years since APL was first identified as a distinct sub-type of AML, the use of ATRA-based therapy has transformed PML-RARα associated APL from a deadly to a largely curable disease (Wang and Chen, 2008). The initial step of this journey can be traced back to the 1970s when Fibach and colleagues demonstrated that AML cells could be induced to undergo terminal differentiation (Fibach et al., 1973), thus forming a basis for differentiation therapy (Sachs, 1978). Subsequently, ATRA was found to induce myeloid differentiation in APL cell lines, as well as the HL-60 AML cell line, leading to the introduction of ATRA-based therapy for PML-RARα associated APL in 1985 (Huang et al., 1988). Though ATRA induced complete remission as a single agent (an unprecedented occurrence), all cases eventually relapsed (Wang and Chen, 2008). ATRA-based therapy of APL has subsequently undergone refinements, notably the inclusion of As_2_O_3_ and the addition of chemotherapy for high-risk cases. This has led to complete remission rates exceeding 93%, with these patients achieving five-year overall survival rates approaching 100% (Wang and Chen, 2008). ATRA-based therapy, however, has not been successful for non-APL AML despite its efficacy in inducing differentiation and cell death in HL-60 and other AML cell lines (Petrie et al., 2009). We previously showed that aberrant histone modifications present on differentiation pathway genes play a critical role in the lack of response and that epigenetic therapy with an inhibitor of LSD1 could reprogram AML cells to respond to ATRA (Glasow et al., 2008; Schenk et al., 2012). While one of the principal reasons underpinning the success of this approach is that LSD1 plays a critical role in the regulation of expression of genes involved in the differentiation of hematopoietic stem cells (Kerenyi et al., 2013), it is noteworthy that a non-canonical scaffolding role for LSD1 has also been identified in APL (Ravasio et al., 2020). Recent research has demonstrated that EZH2 also performs canonical as well as potentially opposing non-canonical roles in multiple types of cancer (Wang and Wang, 2020; Anwar et al., 2021; Huang et al., 2021; Wang et al., 2022a; Wang et al., 2022b; Poplineau et al., 2022), and our results indicate that this is also true in the context of myeloid differentiation. Our study suggests both anti- and pro-differentiative roles for EZH2 in AML and that SAM-competitive inhibition by GSK-343 interferes with both, thus impeding differentiation by ATRA. By contrast, dual inhibition of EZH2 and G9A/GLP histone methyltransferases by substrate-competitive HKMTI-1-005 promotes myeloid differentiation of AML cells, potentially by selectively interfering with the canonical PRC2-related activities of EZH2 while permitting non-canonical, pro-differentiative activity. This could account for the opposite effects of GSK-343 and HKMTI-1-005 in stimulating gene expression programs associated with a hematopoietic stem cell phenotype or myeloid cell development, respectively (Figure 6).

In support of our findings, Poplineau and colleagues recently found that in a genetically engineered mouse model (GEMM) of ATRA-resistant PLZF-RARα APL (arising from the variant t(11;17)(q23;q21) translocation), a structural analog of GSK-343 (GSK-126) was ineffective in preventing disease progression or inducing leukemic blast differentiation with or without ATRA (Poplineau et al., 2022). By contrast, they found that *ex vivo* treatment of the PLZF-RARα GEMM bone marrow with MS1943, a novel EZH2-selective degrader (Ma et al., 2020), was able to erase global H3K27^me3^, reduce cell viability, and induce differentiation. Interestingly, while MS1943 displayed little synergism with ATRA *in vitro*, combinatorial treatment led to a significant improvement in leukemic stem/progenitor cell survival when cells were transplanted into recipient mice. These results potentially point to different roles for EZH2 in the promotion of ATRA-mediated differentiation *versus* the survival of leukemic stem/progenitor cells. One of the non-canonical roles that has emerged for EZH2, which may be relevant to this study, is as a transcriptional activator independent of PRC2 and H3K27 methylation. It has recently been reported that EZH2 contains a cryptic transactivation domain (TAD) that mediates direct interactions with p300 (EP300) and MYC (Jiao et al., 2020; Wang et al., 2022b), as well as another member of the nuclear receptor superfamily, androgen receptor (AR) (Wang et al., 2022a). p300 is an important mediator of ATRA-induced, RXR/RARα-mediated transcriptional activation (Blobel, 2000), functioning both as an acetyltransferase and a scaffolding protein via direct interaction with the RXR/RARα heterodimer (Chan and La Thangue, 2001). This introduces an intriguing possibility that EZH2 function is “biphasic” with regard to transcriptional regulation by RXR/RARα, a mode of action previously postulated for the Jumonji family histone lysine demethylase KDM5B (PLU-1/JARID1B) (Zhang et al., 2014). In the absence of ATRA, KDM5B serves as a bridge between RARα and the SUZ12 component of PRC2, contributing to gene repression. However, in response to ATRA signaling KDM5B functions as a co-activator, possibly contributing to coregulator exchange and unloading of the PRC2 complex. Our finding that RARα/EZH2 interactions increase in response to ATRA treatment supports this notion (Figure 7). Additional research is required, but an intriguing possibility supported by our data is that SAM-competitive inhibitors such as GSK-343 and UNC1999 interfere with the transactivation function of EZH2 in myeloid differentiation, either through inducing a conformational change in EZH2 or due to the presence of a solvent-exposed tail among this class of inhibitors (Bratkowski et al., 2018).

The premise of this study was to identify whether inhibition of EZH2 could be useful as a component of ATRA-based differentiation therapy of AML. However, given the dual inhibitory activity of HKMTI-1-005, the contribution of inhibiting G9A/GLP in addition to EZH2 must be addressed. There is emerging evidence of G9A and GLP playing roles in cancer stemness, metastasis and drug resistance (Haebe et al., 2021; Nachiyappan et al., 2022), as well as having a specific roles in AML (Goyama et al., 2010; Son et al., 2012; Lehnertz et al., 2014). We found that at GI_50_ concentrations, inhibition of G9A/GLP by BIX-01294 in HL-60 cells was not as effective in inducing expression of CD11b as HKMTI-1-005 when used in combination with ATRA. While this further supports a role for EZH2 in myeloid differentiation, the importance of the contribution of the inhibitory activity against G9A/GLP by HKMTI-1-005 in this context remains to be evaluated, particularly that G9A/GLP has a role in mediating silencing by PRC2 (Mozzetta et al., 2014). Recent research has identified a resistance mechanism in AML stem/progenitor cells to G9A/GLP inhibition with BIX-01294, and HKMTI-1-005 should be investigated in this context (Jang et al., 2020). Given the canonical and non-canonical pleiotropic effects of EZH2, inhibition with HKMTI-1-005, either alone or as part of a combinatorial approach, may provide a benefit to the treatment of other cancers. In support of this notion, HKMTI-1-005 has displayed efficacy in breast cancer cell lines (Curry et al., 2015), and more recently in ovarian high-grade serous carcinoma (Spiliopoulou et al., 2022). Here, Spiliopolou and colleagues found that simultaneous inhibition of EZH2 and G9A/GLP by HKMTI-1-005 induced epigenetic changes resulting in the increased expression of immunostimulatory genes. This led to activation of the CXCL10-CXCR3 chemotactic chemokine axis and promotion of an antitumor immune response, while suppressing the tumor-promoting FoxP3+ CD4+ T cells. The ID8 syngeneic mouse model of ovarian cancer was used to demonstrate the efficacy of HKMTI-1-005 *in vivo*, with treatment significantly reducing tumor burden and improving survival.

Numerous SAM-competitive inhibitors have been developed, many of which have demonstrated encouraging activity *in vitro* (Eich et al., 2020; Zeng et al., 2022). Many of these inhibitors are currently in clinical trials, but the majority have demonstrated limited clinical efficacy. For example, a Phase I trial of GSK-126 (GSK2816126) as a single agent in patients with advanced hematologic and solid tumors (NCT02082977) was terminated due to lack of satisfactory clinical activity (Yap et al., 2019). It is very likely that combination treatment strategies will need to be adopted, rather than inhibition of EZH2 alone, as seen from this study. For example, recent research has identified an association between EZH2 and PARP1 (Sbirkov et al., 2017) that is therapeutically targetable with combination treatment (Yamaguchi et al., 2018), or a novel EZH2/PARP1 inhibitor (Wang et al., 2021). In summary, dual EZH2-G9A/GLP inhibition with HKMTI-1-005 shows promise as an approach to promoting the efficacy of ATRA-based therapy in non-APL AML and warrants further investigation. Lastly, this is the first study to use HKMTI-1-005 in combination treatment and its use should be considered in contexts whereby the combinatorial use of SAM-competitive EZH2 inhibitors is being investigated.

## Data Availability

The data presented in the study are deposited in the Gene Expression Omnibus (GEO) repository, accession number GSE222616.
