# Supplementary material for: Dual inhibition of EZH2 and G9A/GLP histone methyltransferases by HKMTI-1-005 promotes differentiation of acute myeloid leukemia cells
Source: Front Cell Dev Biol. 2023 Mar 23;11:1076458. doi: 10.3389/fcell.2023.1076458 (PMC10076884; doi:10.3389/fcell.2023.1076458)
Supplement: Supplementary file 1 [file DataSheet1.pdf]

Dual inhibition of EZH2 and G9A/GLP histone methyltransferases by HKMTI-1-005 promotes differentiation of acute myeloid leukemia cells

Supplementary Material

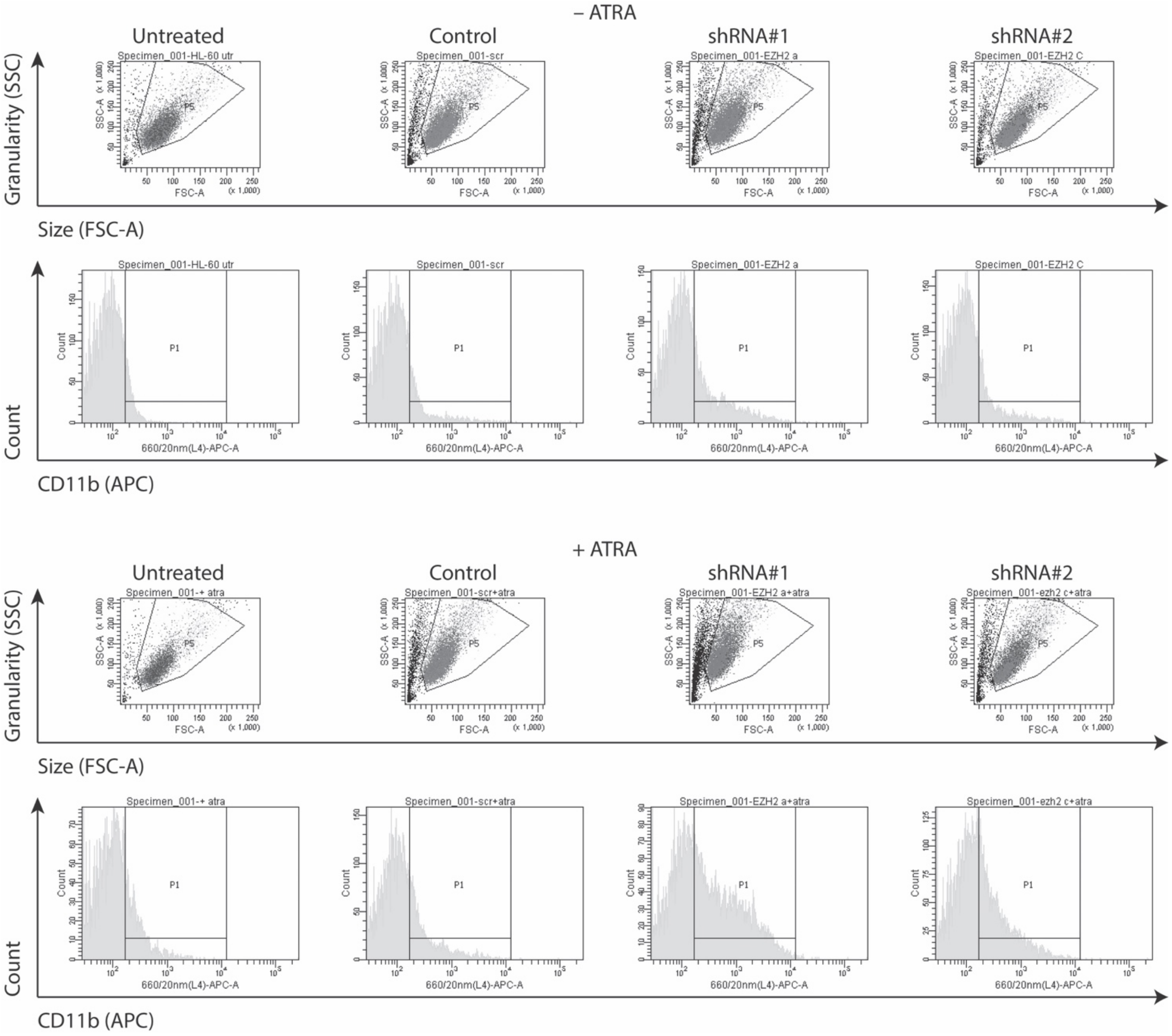

Supplementary Figure 1

Representative flow cytometry plots following *EZH2* KD and ATRA treatment in HL-60. Following *EZH2* lentiviral knockdown and treatment with 0.1 µM all-*trans* retinoic acid (+ATRA) or vehicle control (–ATRA) as indicated for 72 h, HL-60 cells were examined initially by side scatter (SSC) area versus forward scatter (FSC) area, with gating on single cells (P5) to eliminate debris and clumped cells from the analysis. Single cells were then examined by CD11b (MAC-1) expression, gating on CD11b+ cells (P1) using allophycocyanin (APC) conjugated anti-CD11b antibody.

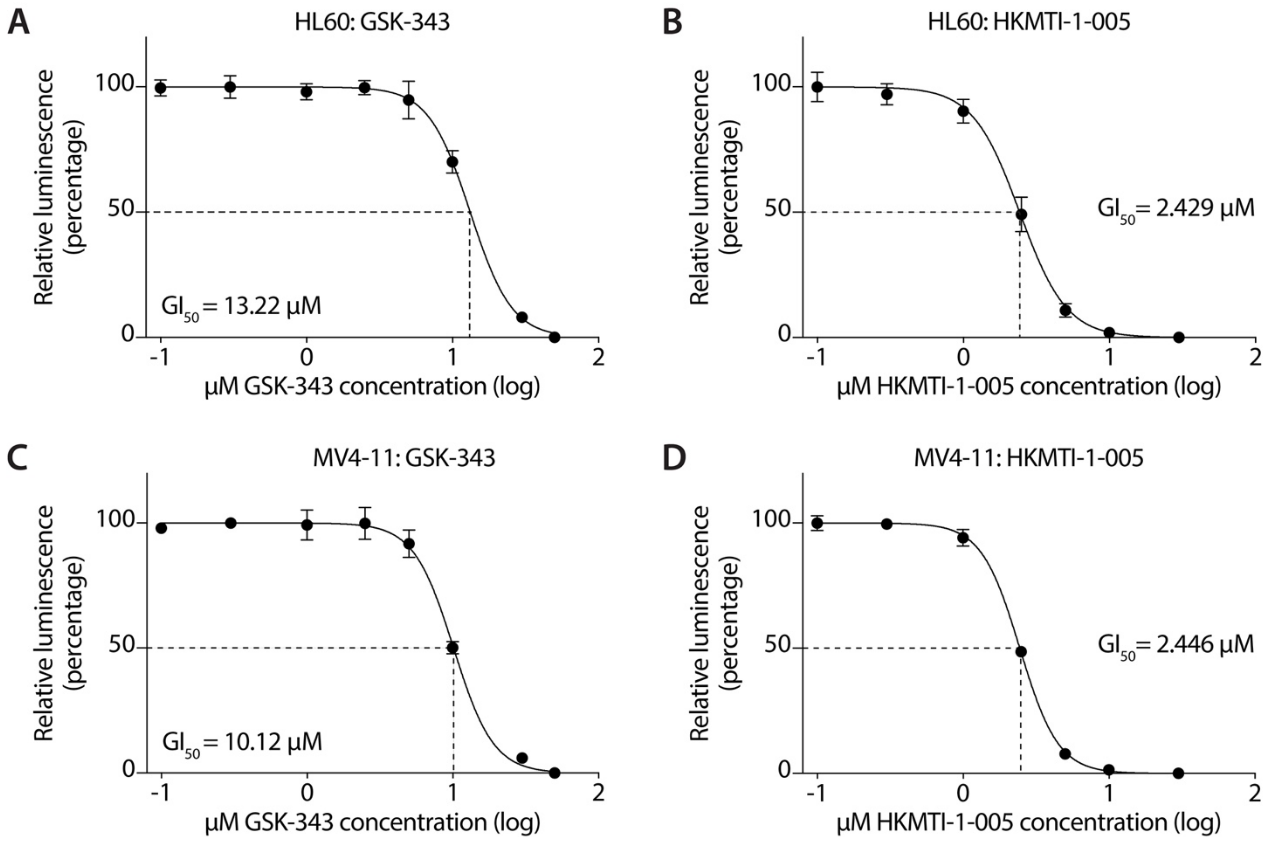

Supplementary Figure 2

Determination of GI_{50} concentrations for GSK-343 and HKMTI-1-005 in HL-60 and MV4-11 cells. HL-60 cells (A) and (B) or MV4-11 cells (C) and (D) were seeded at 20,000 cells per well and treated for 72 h with different concentrations of GSK-343 or HKMTI-1-005 as indicated. Growth inhibitory (GI_{50}) concentrations were determined by measuring cell viability with CellTiter-Glo and generating dose-response curves (GraphPad Prism, version 9.5.0). Values represent the means of three experiments and error bars denote standard deviations.

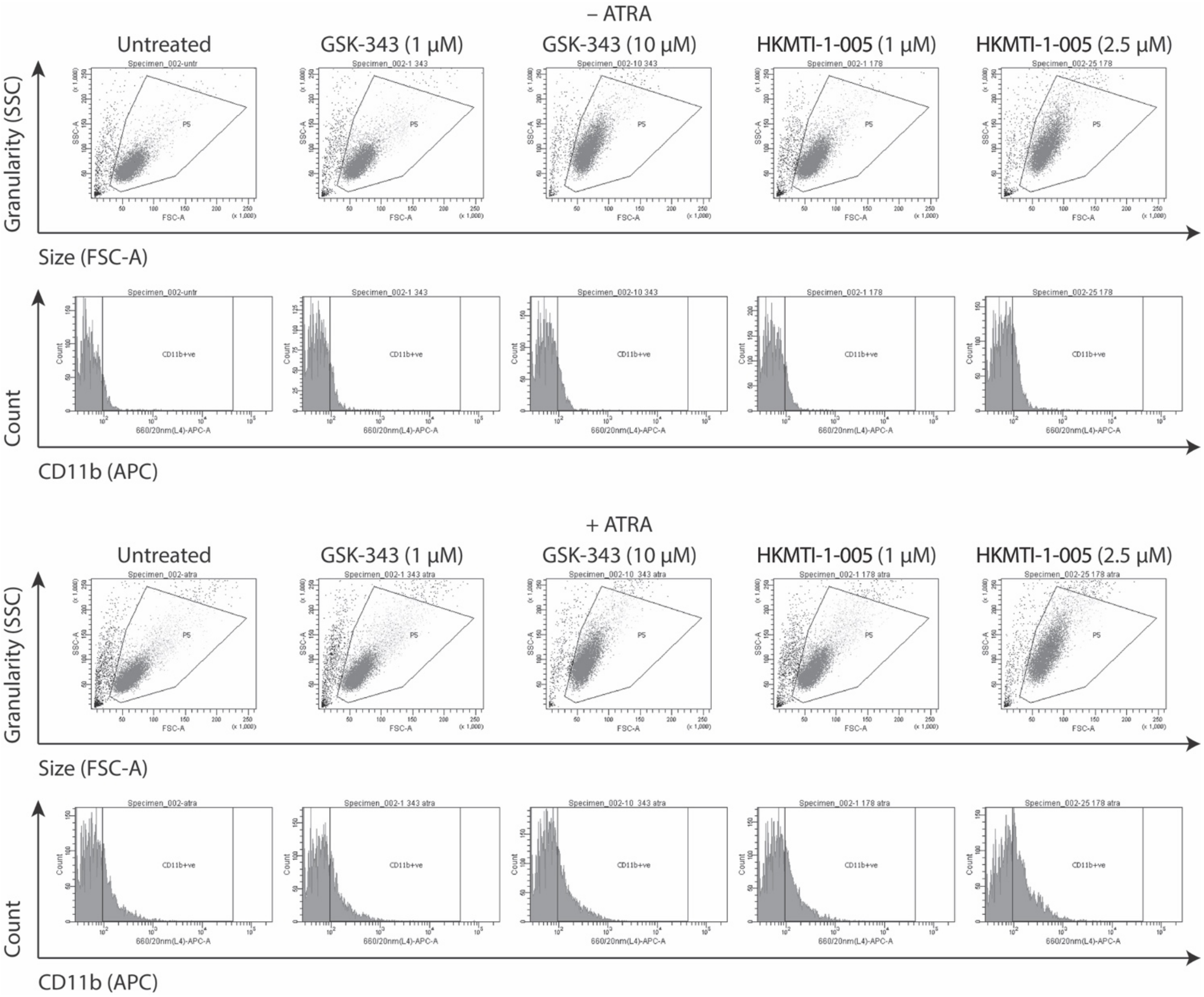

Supplementary Figure 3

Representative flow cytometry plots following treatment of HL-60 cells with GSK-343, HKMTI-1-005 or ATRA. Following treatment with GSK-343 or HKMTI-1-005 at the indicated concentrations plus 0.1 µM all-*trans* retinoic acid (+ATRA) or vehicle control (–ATRA) as indicated for 72 h, HL-60 cells were examined initially by side scatter (SSC) area versus forward scatter (FSC) area, with gating on single cells (P5) to eliminate debris and clumped cells from the analysis. Single cells were then examined by CD11b (MAC-1) expression, gating on CD11b+ cells (P1) using allophycocyanin (APC) conjugated anti-CD11b antibody.

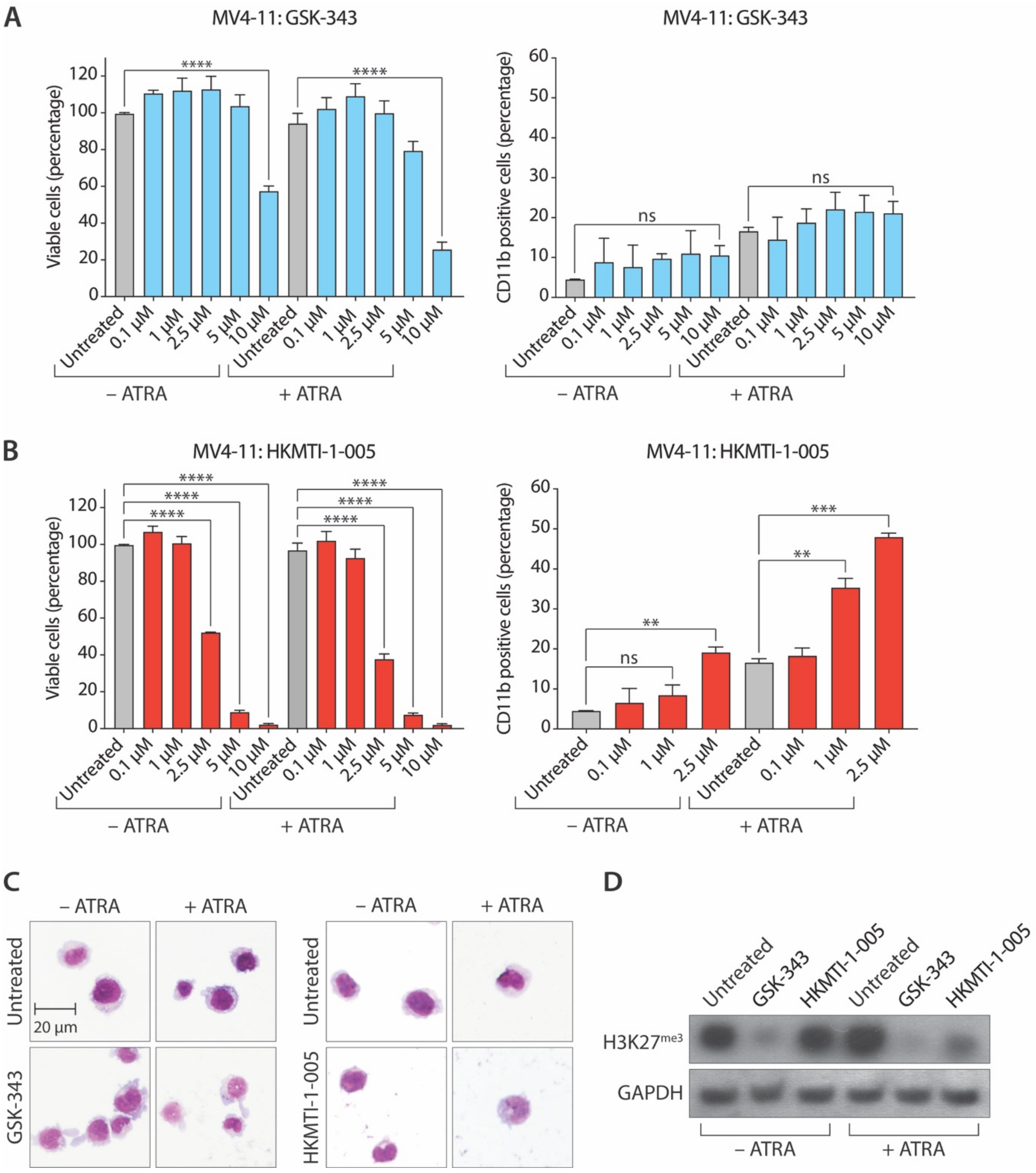

Supplementary Figure 4

Dual inhibition of EZH2-G9A/GLP by HKMTI-1-005 promotes differentiation of MV4-11 AML cells by ATRA. (A) Proportion of live cells as determined by CellTiter-Glo cell viability assay (left panel) and expression of the myeloid differentiation marker CD11b (right panel) following treatment with the indicated concentrations of GSK-343 +/– 0.1 μM ATRA for 72 h. (B) Proportion of live cells as determined by CellTiter-Glo cell viability assay (left panel) and expression of the myeloid differentiation marker CD11b (right panel) following treatment with the indicated concentrations of HKMTI-1-005 +/– 0.1 μM ATRA for 72 h. Values represent the means of three experiments (viability assays) or two experiments (CD11b flow cytometry). Error bars denote standard deviations. **, $p <$

0.01; ***, $p < 0.001$, ****, $p < 0.0001$; ns, no statistical significance. (C) Cell morphology of cells analyzed by May-Grünwald Giemsa staining following treatment with 10 µM GSK-343 (left panel) or 2.5 µM HKMTI-1-005 (right panel) for 72 h. Treatments were performed +/– 0.1 µM ATRA. (D) Immunoblot analysis of levels trimethylated lysine 27 on histone H3 ($H3K27^{me3}$) following treatment (72 h) with 10 µM GSK-343 or 2.5 µM HKMTI-1-005 +/– 0.1 µM ATRA as indicated. GAPDH was used as a loading control.

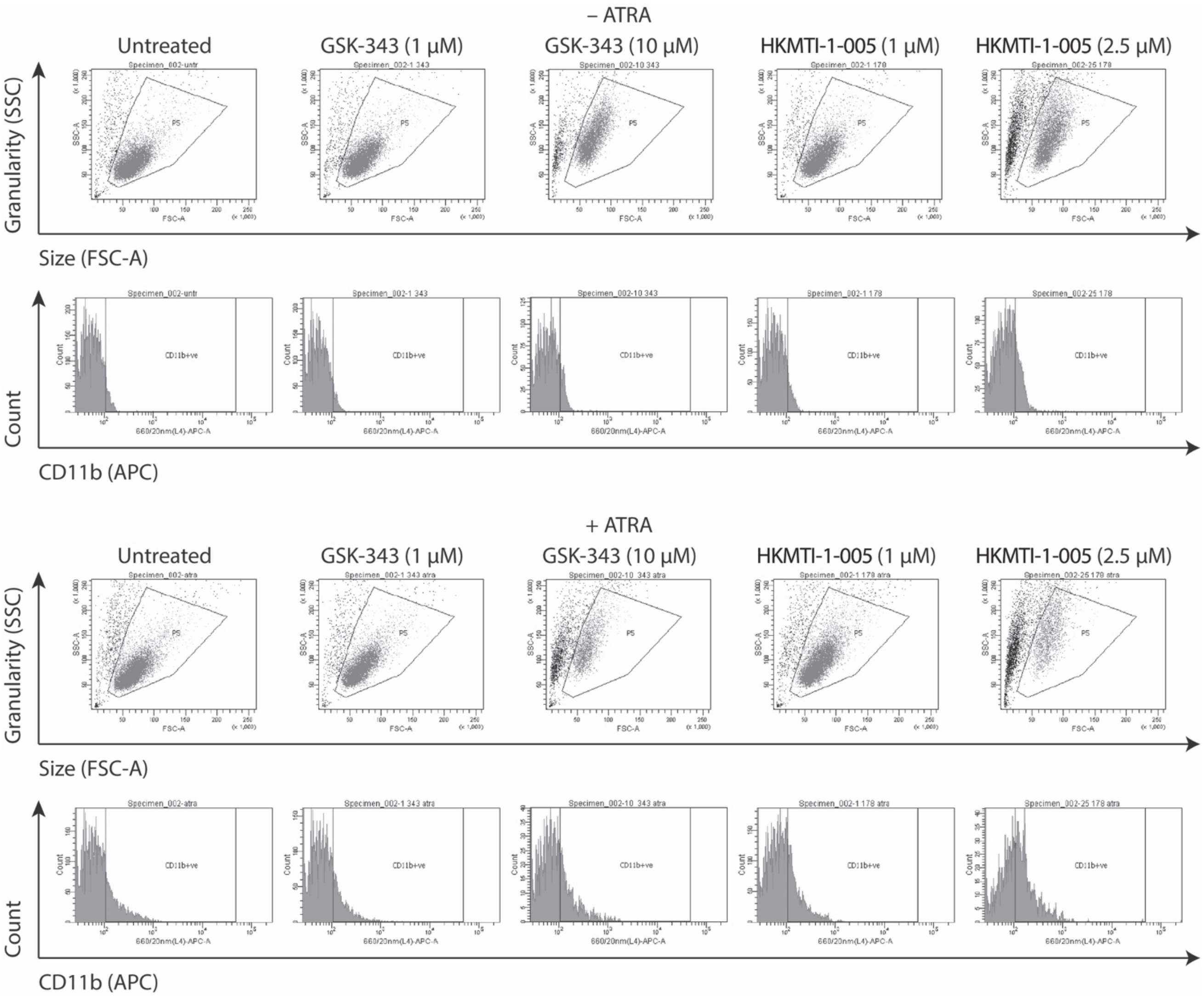

Supplementary Figure 5

Representative flow cytometry plots following treatment of MV4-11 cells with GSK-343, HKMTI-1-005 or ATRA. Following treatment with GSK-343 or HKMTI-1-005 at the indicated concentrations plus 0.1 µM all-*trans* retinoic acid (+ATRA) or vehicle control (–ATRA) as indicated for 72 h, HL-60 cells were examined initially by side scatter (SSC) area versus forward scatter (FSC) area, with gating on single cells (P5) to eliminate debris and clumped cells from the analysis. Single cells were then examined by CD11b (MAC-1) expression, gating on CD11b+ cells (P1) using allophycocyanin (APC) conjugated anti-CD11b antibody.

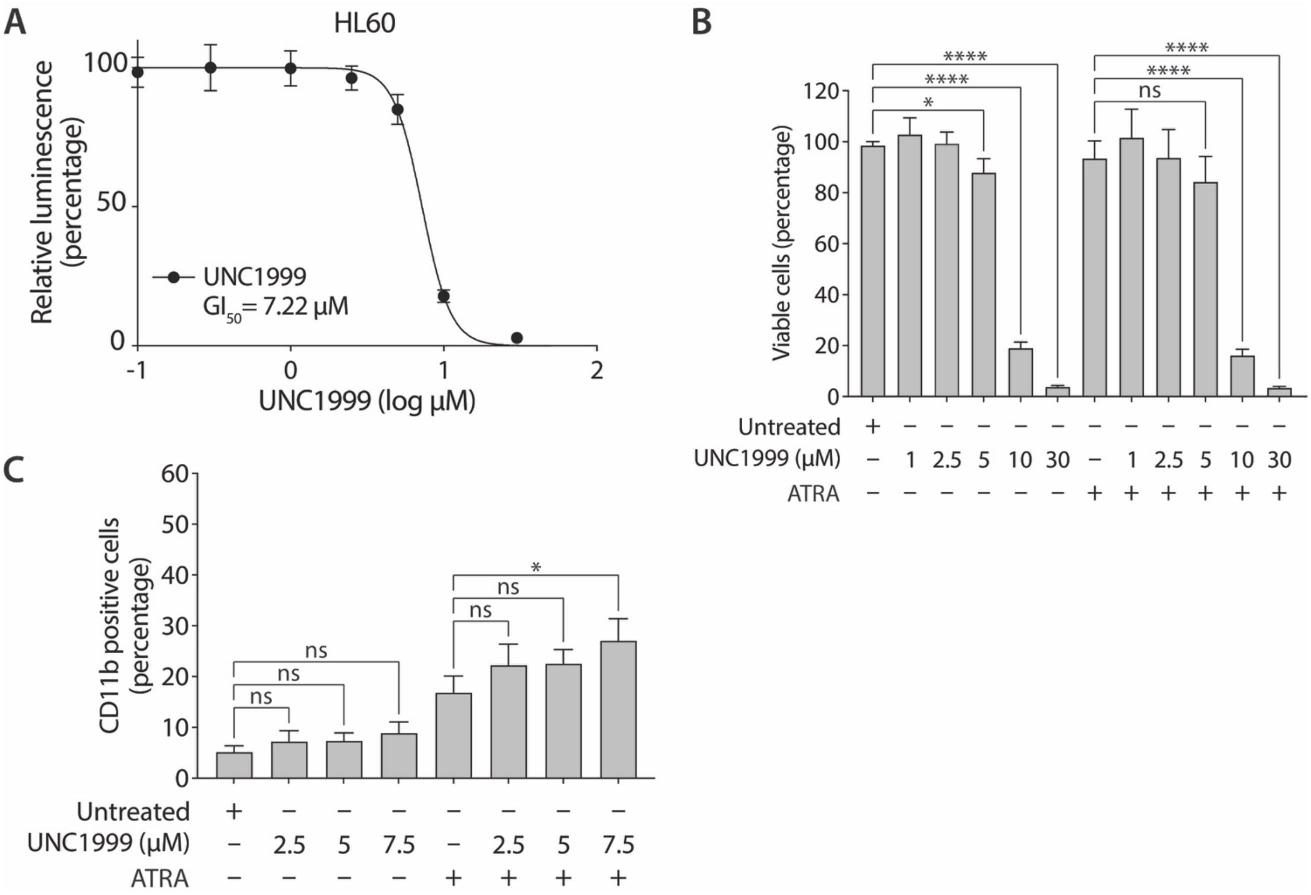

Supplementary Figure 6

Inhibition of EZH1/2 by UNC1999 does not promote differentiation in HL-60 cells. (A) Determination of GI_{50} concentration for UNC1999 in HL-60 cells. Cells were seeded at 20,000 cells per well and treated for 72 h with different concentrations of UNC1999 as indicated. The growth inhibitory (GI_{50}) concentration was determined by measuring cell viability with CellTiter-Glo and generating a dose-response curve (GraphPad Prism, version 9.5.0). (B) Proportion of live cells as determined by CellTiter-Glo cell viability assay following treatment with the indicated concentrations of UNC1999 +/– 0.1 μM ATRA for 72 h. (C) Flow cytometry quantification of the percentage of CD11b positive cells after treatment with 7.5 μM UNC1999 +/– 0.1 μM ATRA for 72 h. Values represent the means of three experiments and error bars denote standard deviations. *, $p < 0.05$; ****, $p < 0.0001$; ns, no statistical significance.

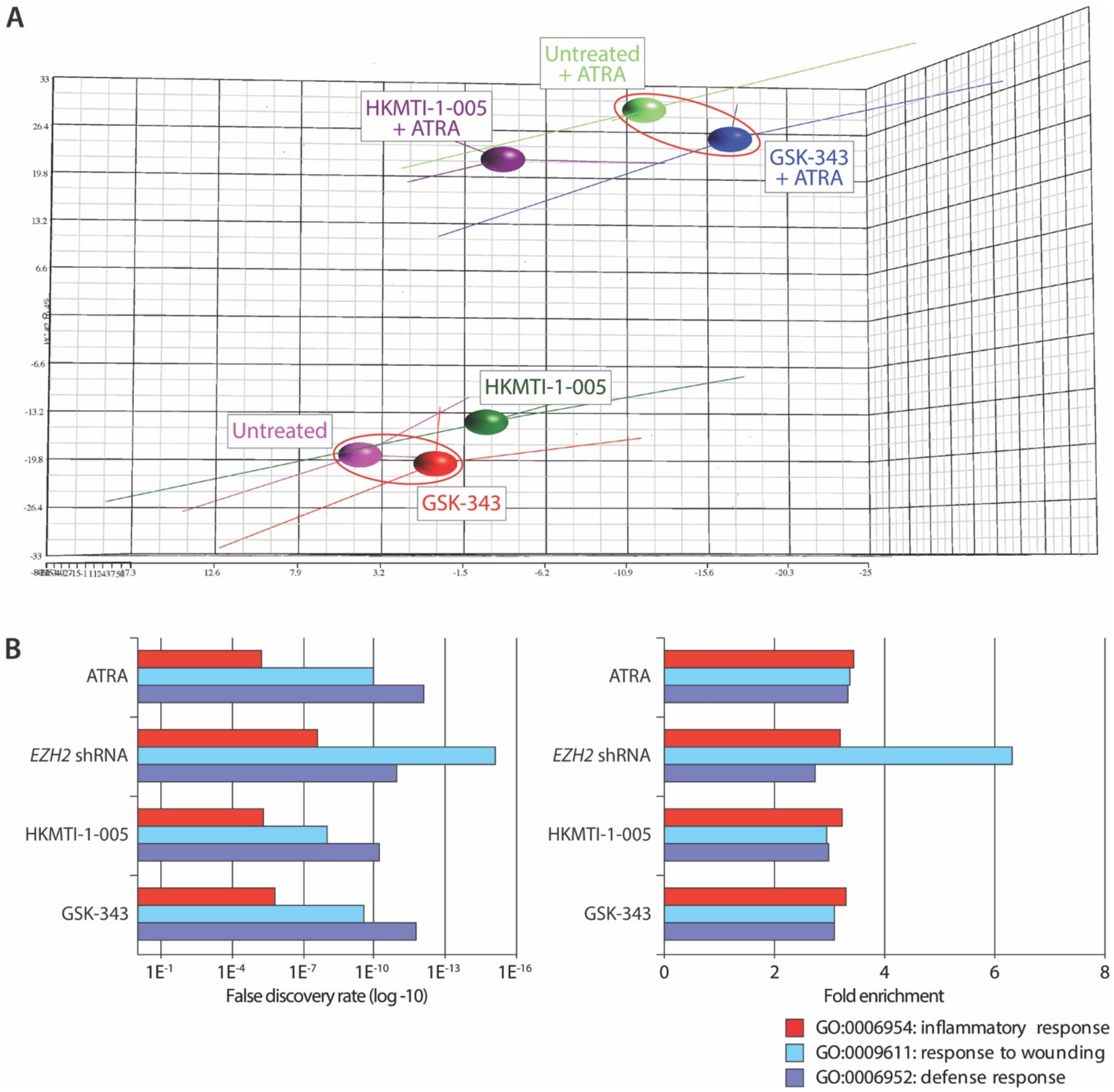

Supplementary Figure 7

Differential gene expression following *EZH2* knockdown or inhibition with GSK-343 or HKMTI-1-005 in HL-60 AML cells. (A) PCA comparing transcriptional programs after EZH2 inhibition in HL-60 cells. PCA of HL-60 cells treated for 72 h with inhibitors (10 µM GSK-343, 2.5 µM HKMTI-1-005) +/– ATRA (0.1 µM) showing similarity between GSK-343 and untreated control samples and separation of HKMTI-1-005-treated cells. Red circles were used to highlight specific grouping of samples of interest. (B) Gene ontology (GO) of upregulated genes in the presence of ATRA. GO analysis of upregulated genes following EZH2 knockdown or drug treatment as annotated (0.1. µM ATRA, 10 µM GSK-343, 2.5 µM HKMTI-1-005 for 72 h) in the presence of 0.1 µM ATRA (72 h) showing enrichment score (left panel) and fold-enrichment over control (right panel) of genes belonging to categories that are connected to myeloid maturation. All conditions in the presence of ATRA exhibit similar enrichment for the expression of genes belonging to inflammatory response, response to wounding and defence response.

Supplementary Tables

Supplementary Table 1: Genes modulated by GSK-343 or GSK-343 plus ATRA

– ATRA

GO Category	Gene List
**Upregulated Genes**	
0050864: regulation of B cell activation	*HMGB3, IL27RA, IL13RA1*
0051249: regulation of lymphocyte activation	*HMGB3, IL27RA, IL13RA1, ICOSLG*
Transcription regulation	*PLAGL1, ZMYND11, SATB1, PCGF2, NOTCH1, PBX3, CRY1*
**Downregulated Genes**	
0002520: immune system development	*EBP, FLT3, SWAP70, FUT10, IFI16, CD74, SYK*
0030097: hemopoiesis	*EBP, FLT3, FUT10, IFI16, CD74, SYK*
0006952: defense response	*IL6R, TLR7, CD74, S100A12*
Transcription	*TCERG1, ZNF217, ZNF296, HIRA, DENND4A, ZNF619, MAML3, IFI16, POLR3E, TTF2*

+ ATRA

GO Category	Gene List
**Upregulated Genes**	
Wnt signaling pathway	*LEF1, FZD3, BCL9*
Regulation of transcription, DNA-dependent	*CEBPA, GATA2, SATB1, RPS14, LEF1, ABCA2, NFE2L3, MLLT3*
Zinc-finger	*GATA2, SMYD2, MYO9A*
**Downregulated Genes**	
Receptor	*FCER2, GPR65, SLAMF7*

Supplementary Table 2: Genes modulated by HKMTI-005/*EZH2* shRNA or HKMTI-1-005/*EZH2* shRNA plus ATRA

– ATRA

GO Category	Gene List
**Upregulated Genes**	
0006119: oxidative phosphorylation	*NDUFB3, NDUFA4, ATP6V1A, UQCR10, UQCR11, ATP6V0A1, ATP6V0D1*
0030097: hemopoiesis	*ROGDI, ADD1, IL31RA*
0006954: inflammatory response	*MYD88, TFRC, IL10RB, CD180, IGFBP4*
0006952: defense response	*MYD88, TFRC, IL10RB, CX3CR1, CD180, IGFBP4, IL31RA*
**Downregulated Genes**	
Hsa05221: acute myeloid leukemia	*CCND1, LEF1, AKT3*
0030097: hemopoiesis	*VEGFA, JAG2, TCEA1*
Developmental protein	*FOXA1, VEGFA, JAG2, SCMH1, TRAF4, ZIC2*
hsa04310: Wnt signaling pathway	*CCND1, CCND2, CSNK1E, LEF1*
0045449: regulation of transcription	*AKNA, BATF3, NFE4, ZNF280B, ZMYM2, FOSL2, FOXA1, LEF1, CBX2, ABCA2, NFXL1, SFMBT2, FLNA, ZNF251, ZNF331, GATA2, MNAT1, SND1, VEGFA, ZNF550, TCEA1, ZNF420, NFE2L3, SCMH1*

+ ATRA

GO Category	Gene List
**Upregulated Genes**	
Hsa04640: Hematopoietic cell lineage	*CD36, TFRC, ITGAM*
0009611: response to wounding	*CD36, TFRC, C3, S100A9, SPP1*
0006952: defense response	*TFRC, C3, S100A9, HP, SPP1*
0006954: inflammatory response	*TFRC, C3, S100A9, SPP1*
**Downregulated Genes**	
Zinc-finger	*KDM7A, ZNF529, FHL1, ZFP30, RBCK1, ZNF141, ZNF8, ZNF253, ZFAND1, ZNF766, ZNF37A*
